# Proteomic analysis by iTRAQ-MRM of soybean resistance to *Lamprosema Indicate*

**DOI:** 10.1186/s12864-017-3825-0

**Published:** 2017-06-06

**Authors:** Weiying Zeng, Zudong Sun, Zhaoyan Cai, Huaizhu Chen, Zhenguang Lai, Shouzhen Yang, Xiangmin Tang

**Affiliations:** Guangxi Academy of Agricultural Sciences, Nanning, Guangxi 530007 China

**Keywords:** Soybean, *Lamprosema indicate* (Fabricius), iTRAQ, MRM, Differentially expressed protein

## Abstract

**Background:**

L*amprosema indicate* is a major leaf feeding insect pest to soybean, which has caused serious yield losses in central and southern China. To explore the defense mechanisms of soybean resistance to *Lamprosema indicate,* a highly resistant line (Gantai-2-2) and a highly susceptible line (Wan 82–178) were exposed to *Lamprosema indicate* larval feedings for 0 h and 48 h, and the differential proteomic analyses of these two lines were carried out.

**Results:**

The results showed that 31 differentially expressed proteins (DEPs) were identified in the Gantai-2-2 when comparing 48 h feeding with 0 h feeding, and 53 DEPs were identified in the Wan 82–178. 28 DEPs were identified when comparing Gantai-2-2 with Wan 82–178 at 0 h feeding. The bioinformatic analysis results showed that most of the DEPs were associated with ribosome, linoleic acid metabolism, flavonoid biosynthesis, phenylpropanoid biosynthesis, peroxisome, stilbenoid, diarylheptanoid and gingerol biosynthesis, glutathione metabolism, pant hormone signal transduction, and flavone and flavonol biosynthesis, as well as other resistance related metabolic pathways. The MRM analysis showed that the iTRAQ results were reliable.

**Conclusions:**

According to the analysis of the DEPs results, the soybean defended or resisted the *Lamprosema indicate* damage by the induction of a synthesis of anti-digestive proteins which inhibit the growth and development of insects, reactive oxygen species scavenging, signaling pathways, secondary metabolites synthesis, and so on.

**Electronic supplementary material:**

The online version of this article (doi:10.1186/s12864-017-3825-0) contains supplementary material, which is available to authorized users.

## Background

Due to the reproduction, nutrition, proliferation, and protection needs between insects and plants, an interactive relationship has been established. Plants also produce constitutive and inducible defense mechanisms. The constitutive defense mechanisms refer to the plants’ own existence materials which inhibit harmful organism infestations [1]. The induced defense mechanisms reflect a type of special insect resistance characteristic when herbivorous insects infect plants [2]. It is one of the important defensive measures by which to explore the regulation mechanisms of plant resistance to pest under the condition of pest persecution at the protein level, such as soybean response to *Prodenia litura* [3], rice response to Brown Planthopper (BPH) [4–6], and *Arabidopsis thaliana* response to *Plutella xylostella* [7, 8], the research of all of which has achieved various degrees of conclusive results.

*Lamprosema indicate* (Fabricius) belongs to the Lepidoptera and Pyralidae groups. It is an important soybean leaf feeding pest, whose larvae lurk inside soybean leaves, cause leaf curling and feed on leaf tissues. This feeding affects the plants’ photosynthesis, and causes abnormal growth [9]. These infestation disasters have occurred in Jilin, southern of Liaoning, and the eastern regions of Sichuan in China. They can occur over multiple generations of plants in 1 year in central and southern China. In the event of serious pest damaged years, only veins and petioles have been left on the blades, thereby causing serious yield losses [10]. Relevant research has been reported in regard to the resource excavations of soybeans’ resistance to *Lamprosema indicata* [10, 11], resistance identification [12, 13], inheritance of the resistance [14, 15] and related gene QTL locations [16, 17]. However, the results of proteomics research which has focused on soybeans’ resistance to *Lamprosema indicata* has not yet been made available.

In this study, soybeans with high resistance and susceptibility to *Lamprosema indicata* were selected as the research objects. The protein expression abundance was analyzed for the soybean following the *Lamprosema indicata* feeding using the iTRAQ-MRM technology. The related proteins’ resistances to *Lamprosema indicate* were identified. The analysis explored how these proteins participated in the responses to resistance, and aimed at resolving the soybeans’ constitutive and inducible defense mechanisms in response to *Lamprosema indicate* at the proteomic level. The results of this study provided a new perspective for the development of the germplasm innovation of soybeans’ resistance to *Lamprosema indicata*, as well as genetic improvement and new species breeding.

## Results

### Basic protein identification information

In this study, iTRAQ technology was used to analyze and compare the different accumulated proteins in different resistant soybean before and after *Lamprosema indicate* feeding. A total of 354,049 spectra were obtained, in which 45,454 spectra were matched to the known soybean spectra in the reference genomes using Mascot software (Matrix Science, London, UK; version 2.3.02). Among these, 28,525 were found to be unique spectra. Here, 15,264 peptides were identified, with 11,068 unique peptides and 4073 proteins (Additional file 1: Table S1and Table S2). The results declared that the iTRAQ has high degree of sensitivity, it can get more comprehensive information than other technique when used to analyze the proteins in plants.

### Whole distribution of the proteomics

**Fig. 1:**
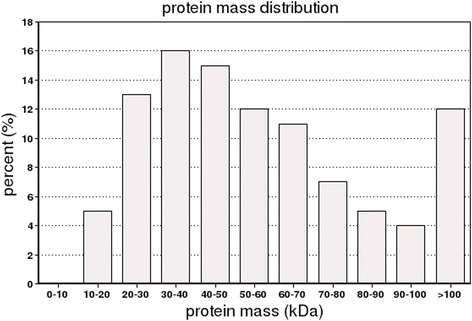
Protein mass distribution. Note: The X axis represents the molecular weights (kDa) of the identified proteins, and the Y axis represents the number of proteins

**Fig. 2:**
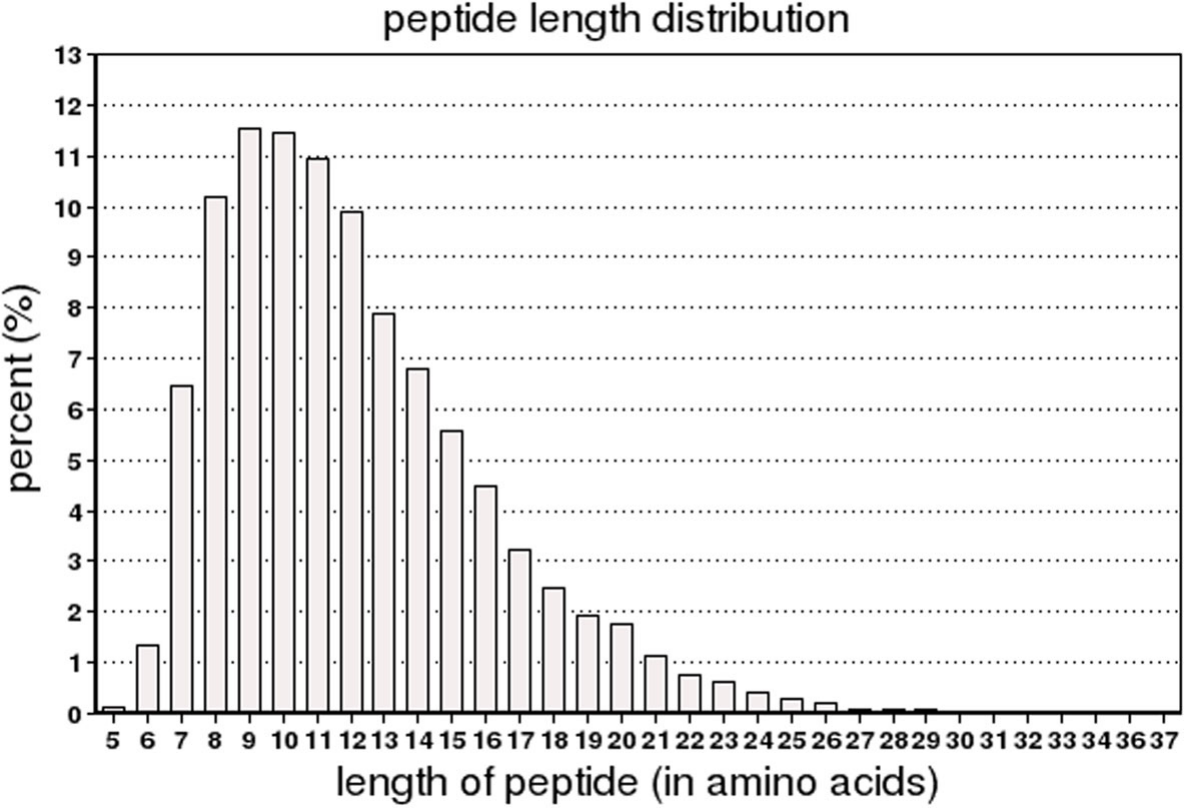
Peptide length distribution. Note: The X axis represents the length of the peptides, and the Y axis represents the number of proteins

**Fig. 3:**
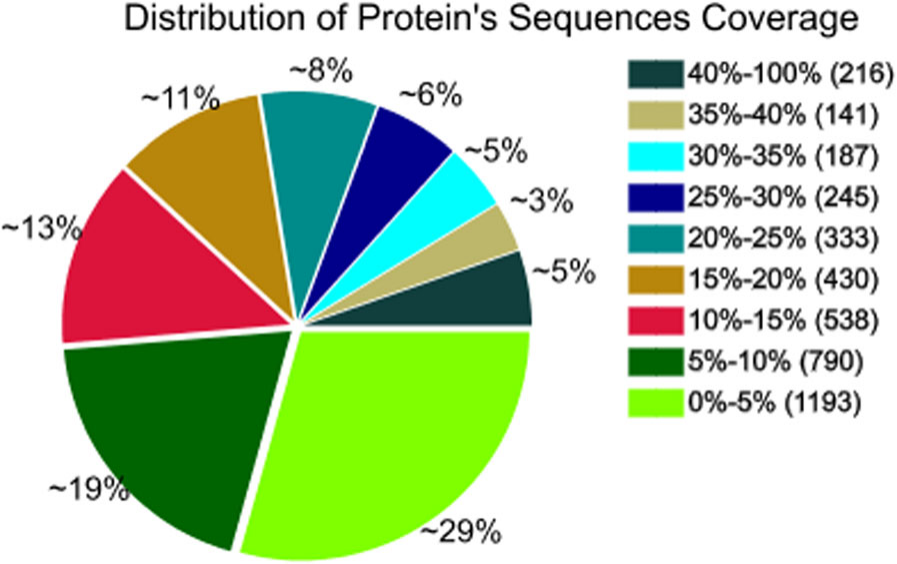
Distribution of the proteins’ sequence coverages. Note: The different colors represent the coverage range of the different sequences, and the pie chart displays the proportion of the number of the different proteins within the scope of coverage in the total protein amount

**Fig. 4:**
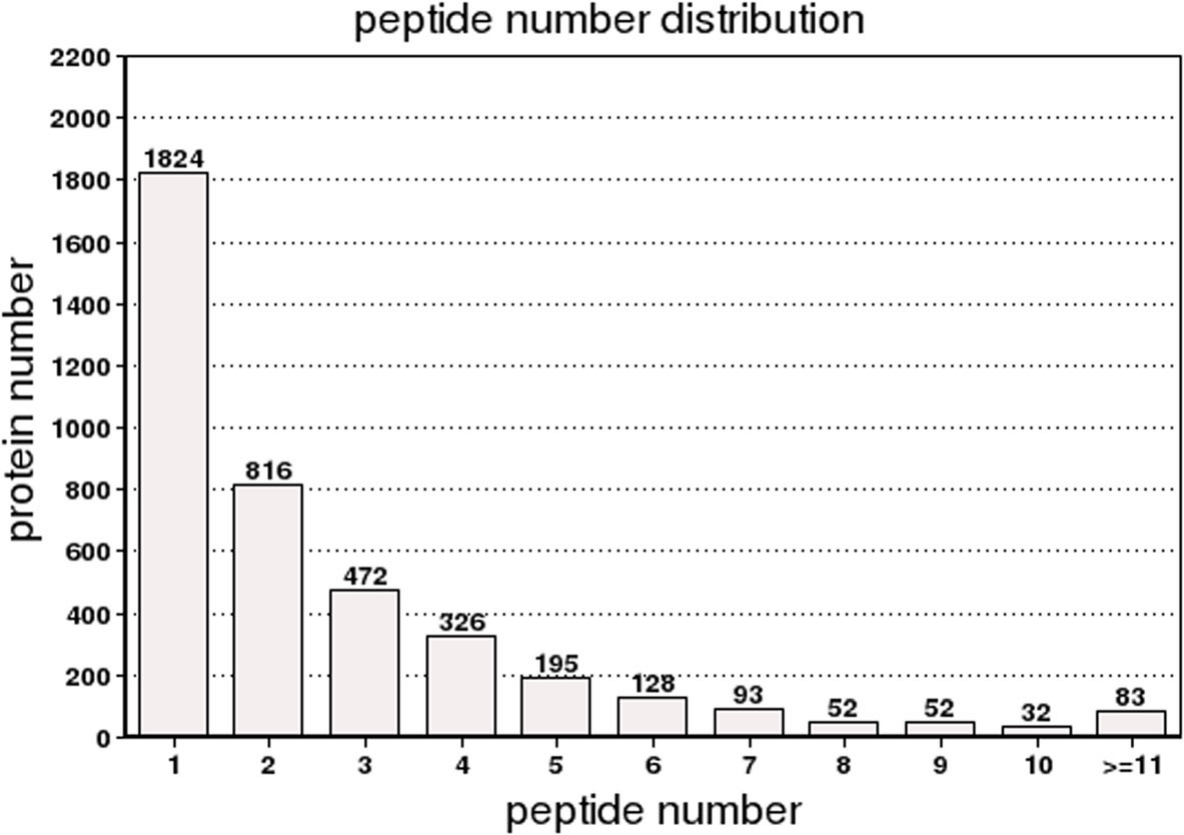
Distribution of the number of peptides. Note: The X axis represents the scope of the number of identified peptides, and the Y axis represents the number of proteins

The statistical analyses of all of the proteins were conducted according to the relative molecular weights (Fig. 1). The results showed that the proteins’ molecular weight distributions which were identified by iTRAQ were relatively broad, and covered the sizes of the different proteins. The analysis of the lengths of all the peptides which were identified by iTRAQ showed that most of the lengths were 7–19 kDa, among which the highest area of distribution was 8–13 kDa (Fig. 2). The distribution of the protein sequence coverage was analyzed for all of the proteins identified by iTRAQ (Fig. 3). The results showed that iTRAQ was able to cover the majority of the expressed proteins. The majority of the identified proteins showed better peptide coverage, of which 52% of the identified proteins had more than 10% of the peptide coverage, and 27% have more than 20% of the peptide coverage. The peptide number analysis for the proteins identified by iTRAQ showed that the peptide segment numbers in most of the proteins were identified to contain less than 10, and the number of proteins decreased with the increased number of matched peptides (Fig. 4). Among these, more than 55.22% (2249/4073) of the protein contained at least two peptide segments, which suggested that the proteins’ isolation and identification were satisfactory. These results indicated that the data qualified for further analysis.

### Repeatability analysis

**Fig. 5:**
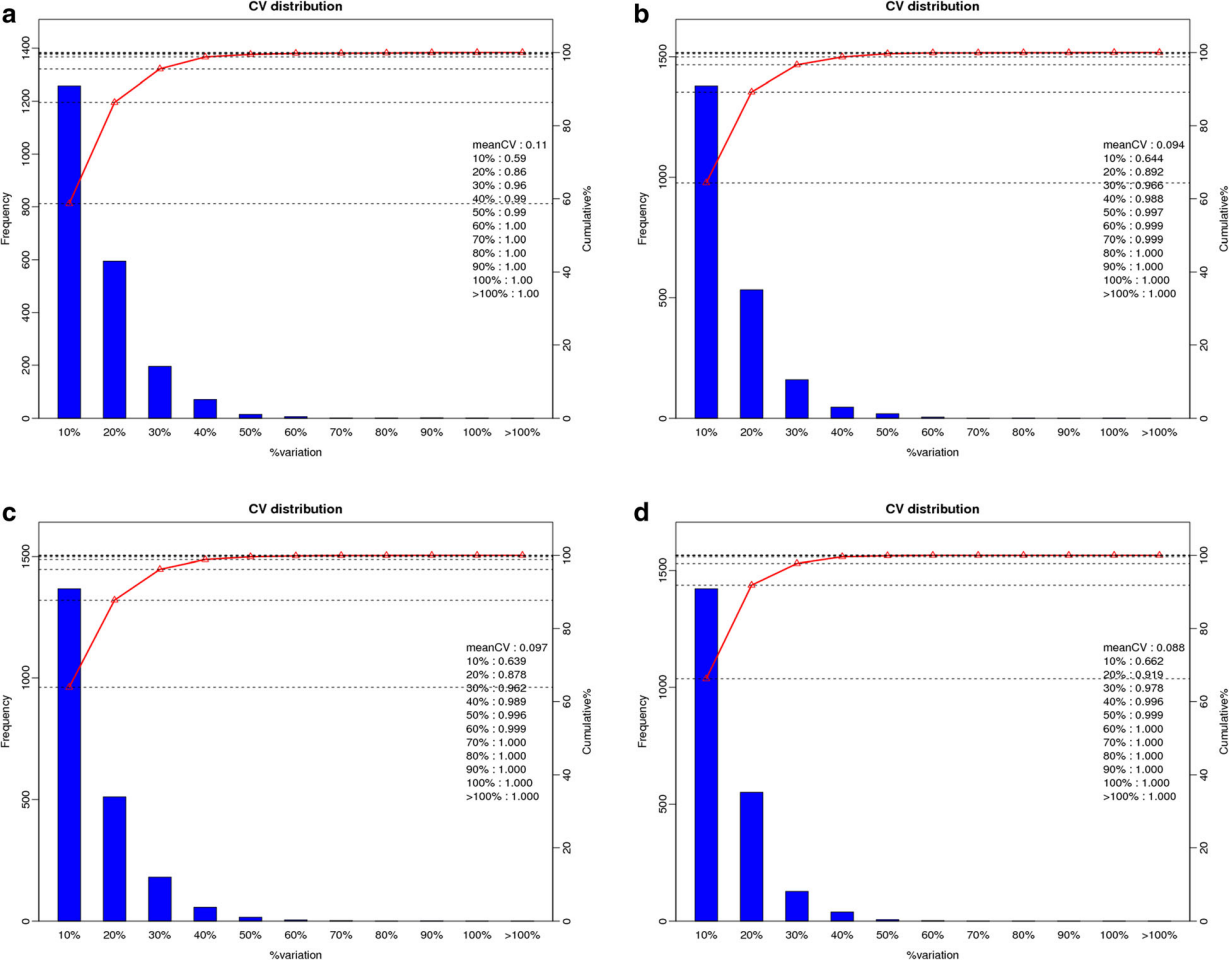
Repeatability analysis. **a**. cv_HR48/HR0_combine, **b**. cv_HS48/HS0_combine, **c**. cv_HR0/HS0_combine, **d**. cv_HR48/HS48_combine. Note: The horizontal axis represents the different levels of variation; the left vertical axis indicates the numbers of the quantitative proteins at different levels of variation and the corresponding column; and the right vertical axis represents the summation of the proportion of the number of proteins at different levels of variation, which accounted for the total number of proteins, along with the corresponding line chart. HR represents the highly resistant line of the Gantai-2-2; HS represents the highly susceptible line of the Wan 82–178; and 0, 48 indicate that the soybean samples were treated with *Lamprosema indicate* at 0 and 48 h, respectively

When the level of variation was 20%, the summation of the proportion of the numbers of proteins at different levels of variation accounted for the total number of proteins reaching more than 0.80, which was considered as high repeatability. In this study, the analysis of two biological replicates in four comparative groups showed that, when the level of variation was 20%, the summations of the proportion of the numbers of proteins at different levels of variation accounted for the total number of proteins were greater than 0.86, indicating that this research showed high repeatability (Fig. 5).

### Analysis of the differentially expressed proteins (DEPs)

A total of 4073 non-redundant proteins were detected by iTRAQ, among which, 3252(79.84%)proteins were quantified (Additional file 1: Table S2). The experimental results showed that 28 DEPs were identified compared Gantai-2-2 (HR) with Wan 82–178 (HS) at 0 h feeding, of which 12 proteins were up-regulated and 16 proteins were down-regulated. Also, 31 DEPs were identified in the Gantai-2-2 compared 48 h feeding with 0 h feeding, including 20 up-regulated proteins and 11 down-regulated proteins. And, 53 DEPs were identified in the Wan 82–178 compared 48 h feeding with 0 h feeding, including 42 up-regulated proteins and 11 down-regulated proteins.

The DEPs in the above three alignment programs were further divided into three classes. The first class was the “DEPs with non-*Lamprosema indicate* induced genotype”, there were 28 DEPs in total. This class of proteins was the “DEPs identified in the Gantai-2-2 compared to the Wan 82–178 before *Lamprosema indicate* feeding induction”, in which four were always up-regulated and three were always down-regulated at 0 h and 48 h. In addition, one protein was up-regulated at 0 h, but down-regulated at 48 h, while the other 20 proteins displayed no changes at 48 h. The second type of proteins were known as the “worm-induced DEPs which appeared in both genotypes”, and included 18 DEPs in total. These mainly presented an up-regulated trend, including 13 proteins which were up-regulated and 5 proteins which were down-regulated. The third class of proteins was the “*Lamprosema indicate*-induced genotype DEPs”, which contained 36 proteins. These proteins appeared only in the highly resistant material or only in the highly susceptible material, including 5 DEPs found only in the Gnatai-2-2 and 31 DEPs found only in the Wan 82–178. In this study, a total of 82 DEPs in the three classes of induced proteins.

A comparison was made of the *Lamprosema indicate* feeding at 0 h and 48 h. The numbers of up-regulated proteins were all greater than the down-regulated proteins in the different resistance materials, which indicated that the *Lamprosema indicate* feeding induced a certain protein expression. This protein expression improved the soybean’s resistance to insects. The total number of DEPs in the highly susceptible material were found to be more than in the highly resistant material, which indicated that it has its own characteristics for highly resistant and highly susceptible soybean in response to *Lamprosema indicate* feeding induced reactions. Also, soybean with different resistant levels may use different defense strategies in response to insects, in which highly susceptible soybean boost more of the proteins involved in the responses to pest stresses.

### DEP cluster analysis

**Fig. 6:**
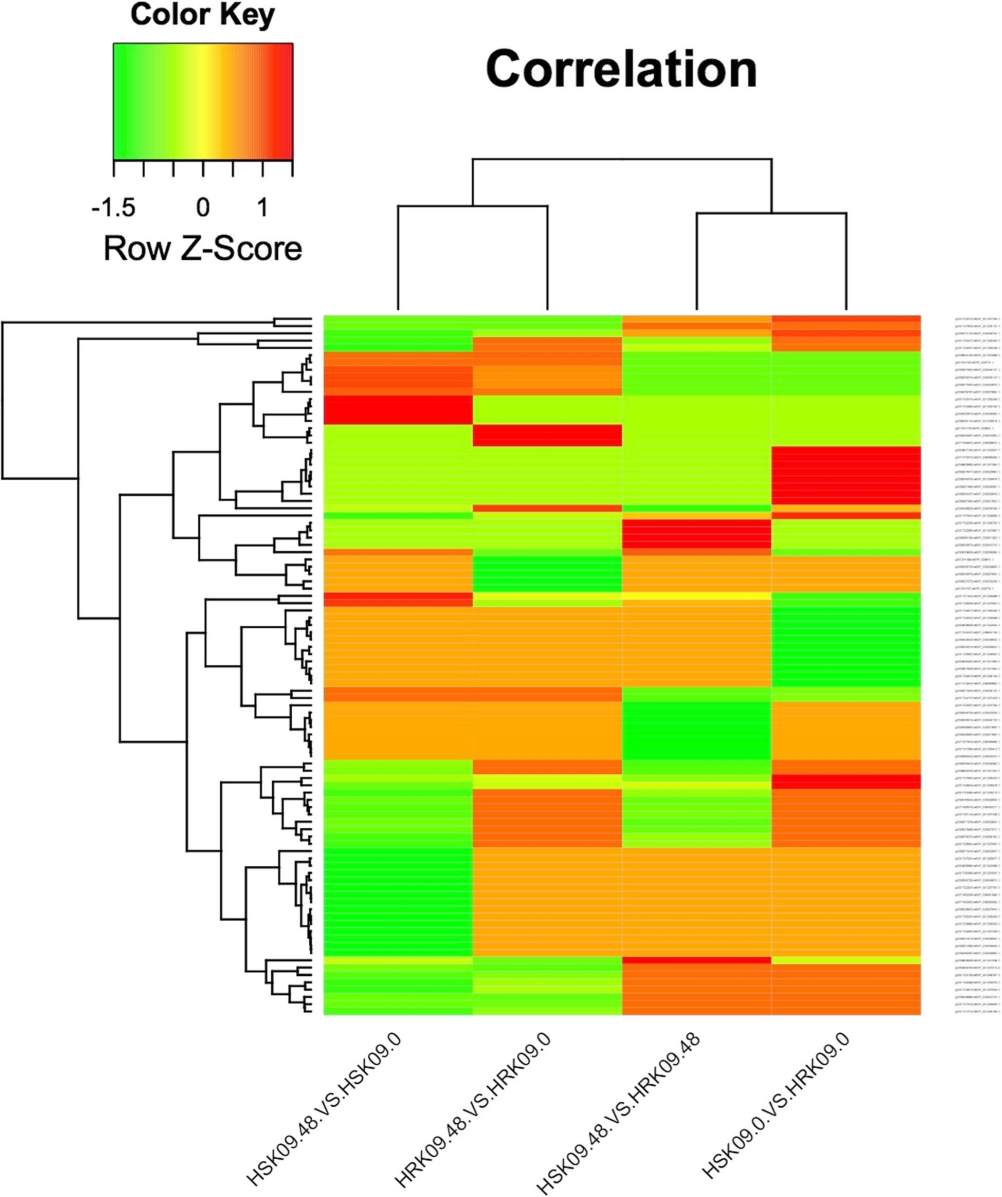
TheDEPs function clustering analysis of four comparison groups. Note: The fold changes of DEPs were presented with different colors, red represents down-regulated; green up-regulated. HR represents the highly resistant line of the Gantai-2-2; HS represents the highly susceptible line of the Wan 82–178; and 0, 48 indicate that the soybean samples were treated with *Lamprosema indicate* at 0 and 48 h, respectively

The DEP clustering results showed that the four comparison groups were divided into two modules (Fig. 6). The first shows that the entire expression styles of HR48/HR0 and HS48/HS0 were similar. The second module shows that the entire expression styles of HR0/HS0 and HR48/HS48 were similar, namely for *Lamprosema indicate* before and after feeding, the expression patterns of the DEPs between two genotypes were similar. These similarities mainly showed that the up- or down-regulation occurred at the same time. This indicated that two kinds of genotypes all exhibited insect-induced DEPs after the *Lamprosema indicate* feeding, which can respond to the stress of insects, and achieve the purpose of defense insects. In addition, the resistant material itself contains a protein which protects against pests.

### Gene ontology (GO) enrichment analysis for all DEPs

**Fig. 7:**
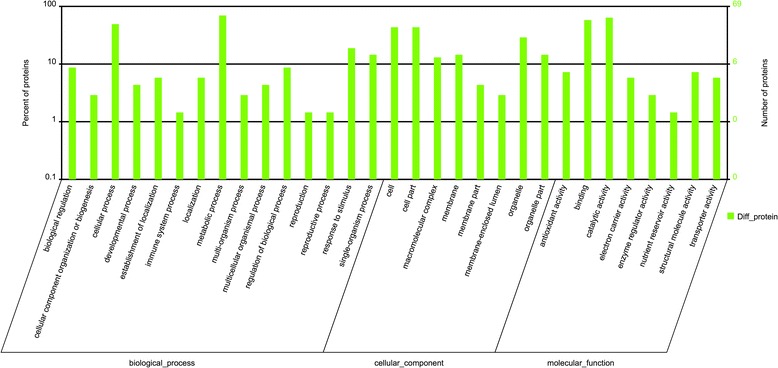
GO enrichment for all of the identified DEPs. Note: The X axis represents each GO term, and the Y axis represents the enrichment ratio of the DEPs in each of the major classes

To further analyze the subcellular localization, molecular functions and biological processes of the DEPs, GO annotation analysis was performed on the 82 DEPs using Blast2go v2.5 software. The results showed that 69 DEPs (84.15%) had been annotated into 31 functional groups, including 15 biological processes, 8 cellular components, and 8 molecular functions (Fig. 7). In the biological processes, the DEPs were mainly involved in metabolic, cellular, responses to stimulus, single-organism processes, and so on. In the cellular components, the DEPs were mainly focused on the cells, cell parts, organelle, membranes, organelle parts, and so on. In the molecular functions, the DEPs were mainly involved in catalytic activities, binding, antioxidant activities and structural molecule activities. These results indicated that the *Lamprosema indicate* response proteins were mainly involved in stress and responses to biotic stimuli, primary metabolic processes, cellular, and so on. We concluded that when the soybean was harmed by the *Lamprosema indicate*, the defense systems in the plants would immediately respond to the stimuli, appropriately increase the metabolic activities in vivo, and produce defense substances, as well as enhancing the activities of various enzymes to promote defense.

### Clusters of Orthologous groups of proteins (COG) function analysis for all of the DEPs

**Fig. 8:**
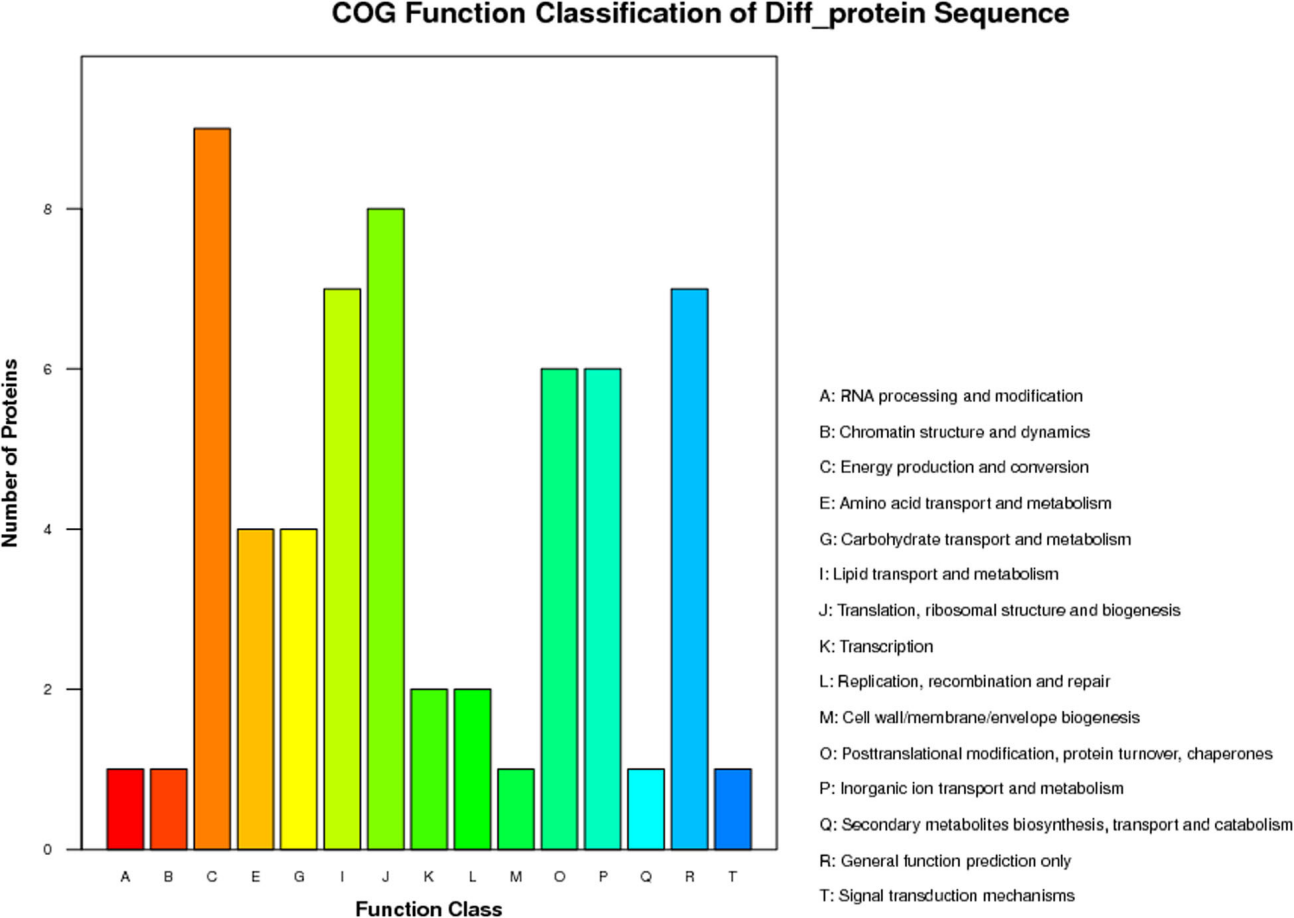
COG functional classes of all of the identified DEPs. Note: The capital letters in the X axis represent the COG categories on the right of column, and the Y axis represents the number of DEPs

A COG enrichment analysis for the 82 DEPs was conducted using Blastx 2.2.24+ software. The results showed that 60 DEPs (73.17%) were annotated to 15 COG categories (Fig. 8), among which six were annotated to more DEPs, including energy production and conversion (C), translation, ribosomal structure and biogenesis (J), general function prediction only (R), lipid transport and metabolism (I), posttranslational modification, protein turnover, chaperones (O), and inorganic ion transport and metabolism (P). The results speculated that most of DEPs related to *Lamprosema indicate* stress were mainly concentrated in these six functional categories. The signal transduction mechanisms (T), secondary metabolites biosynthesis, transport and catabolism (Q), cell wall, membrane and envelope biogenesis (M), RNA processing and modification (A) and chromatin structure and dynamics (B), were annotated to fewer of DEPs. These results indicated that most of the DEPs were contained nearly every aspect of soybean metabolism and growth.

### Kyoto Encyclopedia of genes and genomes (KEGG) enrichment analysis of all of the DEPs

A KEGG pathway enrichment analysis of the 82 DEPs was carried out in a KEGG pathway database using Blast_v2.2.26 software. The results showed that 72 DEPs (87.80%) were annotated to 52 KEGG pathways, and the metabolic pathways (ko01100, 20 DEPs) were the primary pathway enrichment. The second were the biosynthesis of other secondary metabolite (ko01100, 17 DEPs). Based on the KEGG enrichment analysis, it was found that the DEPs participated in the following resistant related pathways, such as ribosome (ko03010, 10 DEPs), linoleic acid metabolism (ko00591, 7 DEPs), flavonoid biosynthesis (ko00941, 6 DEPs), phenylpropanoid biosynthesis (ko00940, 5 DEPs), peroxisome (ko04146, 4 DEPs), stilbenoid, diarylheptanoid, and gingerol biosynthesis (ko00945, 4 DEPs), glutathione metabolism (ko00480, 3 DEPs), pant hormone signal transduction (ko04075, 2 DEPs), and flavone and flavonol biosynthesis (ko00591, 2 DEPs). The pathway analysis showed that the *Lamprosema indicate* induced the DEPs in the metabolic pathways were related to the resistance, they played a defensive role against the insect stress.

**Fig. 9:**
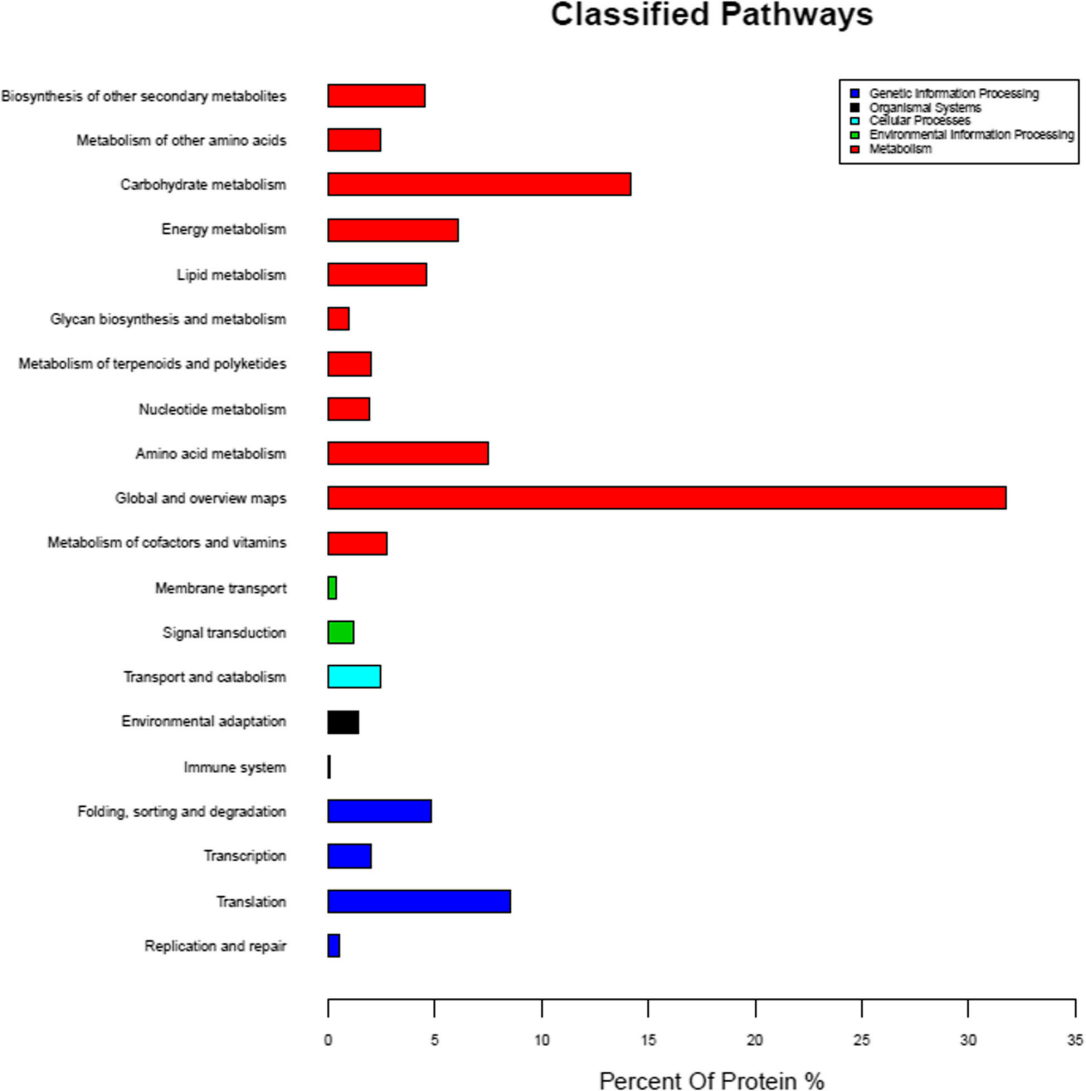
Pathways Classification of all of the identified DEPs. Note: The X axis represent the percent of protein (%), and the Y axis represents the metabolic process

In the KEGG pathway enrichment process, an R language was used for the hyper geometric algorithm. The functions of the DEPs were determined for the classification of the pathways (Table 1, Fig. 9). The DEPs functions were mainly involved in metabolism, including the biosynthesis of other secondary metabolites, along with amino acid, energy, carbohydrate, and lipid metabolisms, and other metabolic processes. The second were genetic information processing, including translation, transcription, folding, sorting and degradation. Other functions, such as signal transduction, transport and catabolism, and environmental adaptation, occupied a lower proportion of the DEPs. There were related to the plants’ components. Also, all of the functional material syntheses in the plants’ were required to be accomplished through metabolic processes. Therefore, a large base existed for these proteins. We concluded that the *Lamprosema indicate* activated both defense and tolerance responses, which in turn made large demands on metabolism. The results provided a basis to better understand the responses which the soybean made under the *Lamprosema indicate* stress conditions.

**Table 1:** Comparison of the DEPs of the Gantai-2-2 and Wan 82–178 after the *Lamprosema indicate* feeding

Accession number	Protein Name forecast	HR48/HR0	HS48/HS0	HR0 /HS0	HR48 /HS48
	Biosynthesis of other secondary metabolite, amino acid metabolism				
gi|363,806,826|ref.|NP_001242544.1|	uncharacterized protein LOC100781477	1.25	0.94	**0.64**	0.82
gi|363,807,958|ref.|NP_001241944.1|	uncharacterized protein LOC100810000	1.32	0.82	**0.56**	1.01
gi|356,571,933|ref.|XP_003554125.1|	PREDICTED: cytochrome P450 82A4-like	1.79	**1.67**	**1.89**	**1.75**
gi|351,726,399|ref.|NP_001237637.1|	isoflavone reductase homolog 2	1.35	**1.75**	0.88	0.69
gi|356,556,726|ref.|XP_003546674.1|	PREDICTED: soyasapogenol B glucuronide galactosyltransferase-like	1.47	**1.82**	0.83	0.78
gi|356,501,269|ref.|XP_003519448.1|	PREDICTED: 1-aminocyclopropane-1-carboxylate oxidase	1.16	**1.54**	1.07	0.72
gi|351,723,089|ref.|NP_001235219.1|	chalcone--flavonone isomerase 1A	1.35	**1.82**	0.84	**0.64**
gi|356,576,075|ref.|XP_003556160.1|	PREDICTED: peroxidase 12-like	1.12	**2.13**	1.05	**0.56**
gi|356,567,390|ref.|XP_003551903.1|	PREDICTED: spermidine hydroxycinnamoyl transferase-like	1.16	0.94	**3.23**	1.90
gi|356,524,057|ref.|XP_003530649.1|	PREDICTED: 3-hydroxyisobutyryl-CoA hydrolase-like protein 3, mitochondrial-like isoform X1	**0.67**	1.05	**1.61**	1.12
gi|351,723,275|ref.|NP_001237785.1|	ascorbate peroxidase 1, cytosolic	**0.63**	0.72	**13.61**	**6.90**
gi|351,727,959|ref.|NP_001235131.1|	OAS-TL4 cysteine synthase	0.82	1.52	**8.02**	**7.63**
gi|351,723,011|ref.|NP_001236240.1|	uncharacterized protein LOC100305498	0.96	**0.59**	0.77	1.24
	Energy and carbohydrate metabolisms				
gi|363,808,320|ref.|NP_001241992.1|	uncharacterized protein LOC100781853 precursor	**1.82**	1.03	**0.51**	0.88
gi|351,727,401|ref.|NP_001238695.1|	uncharacterized protein LOC100499708	0.89	**1.85**	**4.64**	**1.77**
gi|356,507,817|ref.|XP_003522660.1|	PREDICTED: plasma membrane ATPase 4-like isoformX1	0.83	1.15	**1.56**	1.17
gi|91,214,157|ref.|YP_538779.1|	photosystem IIprotein L	**2.38**	**1.92**	0.94	1.06
gi|571,532,037|ref.|XP_006600158.1|	PREDICTED: uncharacterized protein LOC100527923 isoform X1	**1.54**	1.35	**0.62**	0.71
gi|571,486,515|ref.|XP_006590377.1|	PREDICTED: glutamine synthetase cytosolic isozyme 1 isoform X1	1.18	**1.64**	0.92	**0.64**
gi|351,724,891|ref.|XP_001237329.1|	enolase	1.18	**1.56**	0.78	0.92
gi|356,573,103|ref.|XP_003554704.1|	PREDICTED: pro-hevein	**3.85**	**5.88**	0.94	**0.65**
gi|91,214,152|ref.|YP_538774.1|	acetyl-CoA carboxylase carboxyltransferase beta subunit	**0.61**	**0.61**	1.04	1.06
gi|356,499,929|ref.|XP_003518788.1|	PREDICTED: calvin cycle protein CP12–2, chloroplastic	**0.38**	1.20	**1.74**	**0.56**
gi|351,723,155|ref.|NP_001236757.1|	ruBisCO-associated protein	**2.38**	**2.70**	0.83	0.79
gi|356,535,214|ref.|XP_003536143.1|	PREDICTED: rubisco accumulation factor 1, chloroplastic-like	**0.61**	**0.53**	1.01	1.15
	Lipid metabolism				
gi|351,727,981|ref.|NP_001238203.1|	lipoxygenase L-5	**1.54**	**1.64**	0.59	**0.59**
gi|351,726,848|ref.|NP_001238676.1|	seed linoleate 9S–lipoxygenase	**1.61**	**1.89**	0.78	**0.63**
gi|351,727,312|ref.|NP_001238692.1|	lipoxygenase	**1.64**	**1.67**	0.74	0.73
gi|351,725,145|ref.|NP_001237338.1|	lipoxygenase-10	1.30	**1.69**	0.92	**0.62**
gi|356,519,443|ref.|XP_003528382.1|	PREDICTED: linoleate 9S–lipoxygenase 1-like isoform 1	1.16	**2.22**	0.69	**0.37**
gi|356,571,545|ref.|XP_003553937.1|	PREDICTED: alpha-dioxygenase 1-like	1.37	**2.56**	1.05	0.65
gi|363,806,966|ref.|NP_001242568.1|	uncharacterized protein LOC100789930	1.64	**1.96**	1.07	0.90
gi|356,538,921|ref.|XP_003537949.1|	PREDICTED: linoleate 13S–lipoxygenase 2–1, chloroplastic-like	1.09	**1.61**	0.94	0.70
gi|356,520,511|ref.|XP_003528905.1|	PREDICTED: peroxisomal fatty acid beta-oxidation multifunctional protein AIM1-like	0.89	0.78	**0.59**	0.69
gi|351,724,717|ref.|NP_001237323.1|	lipoxygenase-9	1.37	**1.52**	**0.47**	**0.42**
gi|356,571,378|ref.|XP_003553854.1|	PREDICTED: quinone oxidoreductase-like protein At1g23740, chloroplastic-like	1.33	**2.17**	0.78	**0.45**
	Signal transduction				
gi|356,517,686|ref.|XP_003527517.1|	PREDICTED: probable carboxylesterase 15-like	1.27	**1.89**	0.81	**0.53**
gi|356,521,488|ref.|XP_003529387.1|	PREDICTED: probable carboxylesterase 12-like	0.76	1.05	**1.51**	1.20
	Transport and catabolism				
gi|351,721,352|ref.|NP_001238486.1|	superoxide dismutase [Fe], chloroplastic precursor	**1.81**	1.28	**0.36**	**0.56**
gi|351,726,636|ref.|NP_001237901.1|	iron-superoxide dismutase	**2.70**	1.20	**0.27**	**0.65**
gi|356,532,545|ref.|XP_003534832.1|	PREDICTED: phospholipase D p1-like isoform X1	1.19	0.73	**0.62**	1.10
gi|363,806,966|ref.|NP_001242568.1|	uncharacterized protein LOC100789930	**1.64**	**1.96**	1.07	0.90
	Environmental adaptation				
gi|571,484,620|ref.|XP_006589610.1|	PREDICTED: cysteine-rich receptor-like protein kinase 26-like	**0.60**	0.46	1.00	1.30
	Translation				
gi|359,806,656|ref.|NP_001241536.1|	stem 31 kDa glycoprotein precursor	**2.08**	**1.75**	**0.57**	0.87
gi|351,721,272|ref.|NP_001236180.1|	acid phosphatase precursor	**1.61**	**1.85**	1.23	1.08
gi|571,472,621|ref.|XP_006585662.1|	PREDICTED: 60S ribosomal protein L7–4-like	1.30	0.81	**0.54**	0.88
gi|351,724,617|ref.|NP_001236040.1|	uncharacterized protein LOC100500043	1.30	0.72	**0.44**	0.90
gi|351,724,973|ref.|NP_001238100.1|	uncharacterized protein LOC100306646	1.14	0.72	**0.54**	0.85
gi|91,214,176|ref.|YP_538800.1|	ribosomal protein S8	**0.62**	0.79	1.04	0.83
gi|351,723,489|ref.|NP_001235745.1|	uncharacterized protein LOC100305975	0.91	**0.60**	1.01	**1.57**
gi|91,214,188|ref.|YP_538811.1|	ribosomal protein S15	**1.75**	1.25	0.64	0.98
	Folding, sorting, and degradation				
gi|571,460,036|ref.|XP_006581588.1|	REDICTED: protein disulfide-isomerase-like	1.35	**1.69**	1.17	0.88
gi|571,463,350|ref.|XP_006582592.1|	PREDICTED: probable protein disulfide-isomerase A6-like	1.28	**1.69**	1.01	0.76
gi|380,848,783|ref.|NP_001237210.2|	lectin precursor	**2.33**	**2.17**	0.73	0.84
gi|356,531,872|ref.|XP_003534500.1|	PREDICTED: calreticulin-3-like	0.95	**1.56**	1.01	0.68
gi|351,728,052|ref.|NP_001235646.1|	uncharacterized protein LOC100305968	1.43	0.86	**0.66**	1.10
gi|356,520,875|ref.|XP_003529085.1|	PREDICTED: thioredoxin-like 2, chloroplastic-like	0.81	**0.57**	0.81	0.94
	Transcription				
gi|356,538,787|ref.|XP_003537882.1|	PREDICTED: glycine-rich RNA-binding protein 2-like	**0.46**	**0.42**	0.94	1.07
gi|359,806,184|ref.|NP_001240946.1|	uncharacterized protein LOC100812934	**0.56**	**0.60**	1.04	1.07
gi|356,549,367|ref.|XP_003543065.1|	PREDICTED: splicing factor 3B subunit 2-like	**0.64**	1.19	1.34	0.78
gi|351,725,567|ref.|NP_001236841.1|	HMG1/2-like protein	1.15	0.65	**0.57**	1.25
	Other aspects				
gi|358,248,112|ref.|NP_001239816.1|	uncharacterized protein LOC100813859	1.00	**0.62**	1.04	0.96
gi|351,726,088|ref.|NP_001235579.1|	uncharacterized protein LOC100500267 precursor	**1.67**	**2.13**	0.95	0.72
gi|351723671ref|NP_0012375431|	trypsin inhibitor	**1.72**	**2.33**	1.06	0.93
gi|356,527,272|ref.|XP_003532236.1|	trypsin inhibitor A-like	**2.12**	1.41	1.12	**1.52**
gi|351,722,301|ref.|NP_001237751.1|	Kunitz trypsin protease inhibitor-like precursor	1.47	**1.67**	1.26	1.06
gi|356,548,666|ref.|XP_003542721.1|	PREDICTED: triose phosphate/phosphate translocator, chloroplastic	**1.79**	**1.79**	1.06	1.08
gi|356,571,630|ref.|XP_003553979.1|	PREDICTED: light-regulated protein-like	**0.61**	**0.53**	1.37	**1.57**
gi|356,551,590|ref.|XP_003544157.1|	PREDICTED: cucumisin-like isoform X1	**0.63**	**0.57**	1.32	1.42
gi|351,725,047|ref.|NP_001236055.1|	uncharacterized protein LOC547916	1.64	**5.26**	0.83	**0.29**
gi|351,726,694|ref.|NP_001237647.1|	uncharacterized protein LOC100306363	1.45	**2.17**	0.95	**0.57**
gi|359,806,316|ref.|NP_001241224.1|	uncharacterized protein LOC100794293	1.12	**2.44**	0.77	**0.35**
gi|356,516,555|ref.|XP_003526959.1|	PREDICTED: probable inactive purple acid phosphatase 27-like	1.41	**1.67**	0.69	**0.60**
gi|356,539,609|ref.|XP_003538289.1|	PREDICTED: uncharacterized protein LOC100789683	0.48	**0.57**	1.28	**1.84**
gi|358,249,018|ref.|NP_001239979.1|	uncharacterized protein LOC100792638	0.68	0.97	**1.56**	1.16
gi|571,475,075|ref.|XP_006586458.1|	PREDICTED: auxin transport protein BIG-like	0.64	0.82	**1.62**	1.33
gi|363,807,160|ref.|NP_001242601.1|	uncharacterized protein LOC100787890	0.64	0.82	**1.62**	1.33
gi|351,726,331|ref.|NP_001236355.1|	uncharacterized protein LOC100500579 precursor	1.61	**1.59**	1.28	1.22
gi|351,727,321|ref.|NP_001235877.1|	PR-5b protein precursor	1.23	**1.92**	1.01	0.72
gi|351,724,557|ref.|NP_001236038.1|	stress-induced protein SAM22	2.38	**12.50**	1.30	**0.24**
gi|351,725,669|ref.|NP_001235053.1|	glucosyltransferase	1.30	**1.59**	1.25	0.87
gi|356,536,733|ref.|XP_003536890.1|	PREDICTED: histone H1-like	**1.59**	1.00	**0.64**	0.98

HR represents the highly resistant line of the Gantai-2-2; HS represents the highly susceptible line of the Wan 82–178; and 0, 48 indicate that the soybean samples were treated with *Lamprosema indicate* at 0 and 48 h, respectively. The bold highlighted numbers indicate the significant differences found in this type of comparison

### Analysis of DEP interaction network

**Fig. 10:**
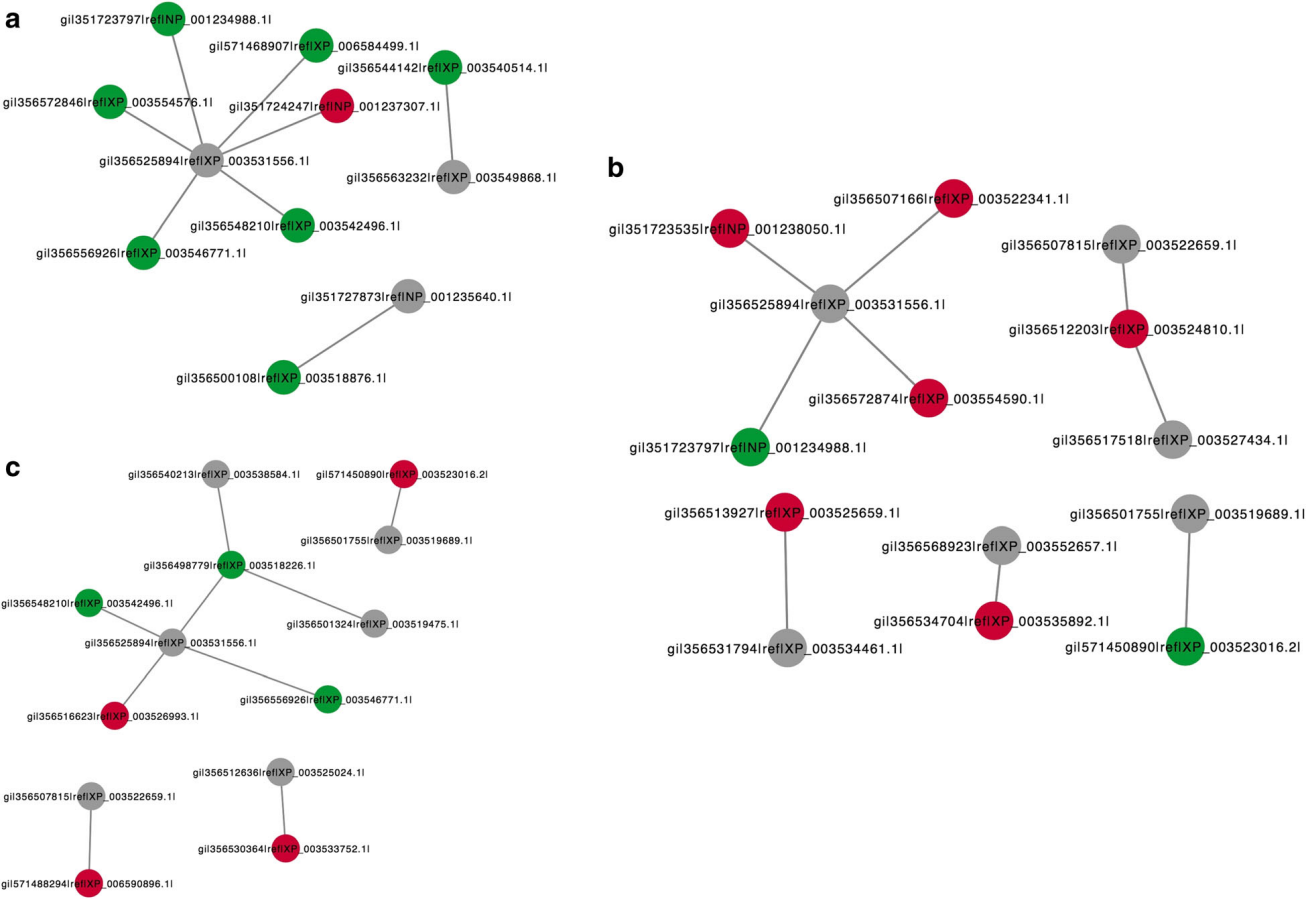
Network metabolic analysis for DEPs. **a**. HR48/HR0, **b**. HS48/HS0, **c**. HS0/HR0. Note: The fold changes of differential expressed proteins were presented with different colors, red represents down-regulated; green up-regulated. HR represents the highly resistant line of the Gantai-2-2; HS represents the highly susceptible line of the Wan 82–178; and 0, 48 indicate that the soybean samples were treated with *Lamprosema indicate* at 0 and 48 h, respectively

The results of the DEP interaction network showed that 48 h after *Lamprosema indicate* feeding stress, compared to 0 h, the interaction network of identified part of the DEPs in the resistant material Guantai-2-2 (HR) was divided into three levels (Fig. 10a), i.e. gi|356,525,894|ref.|XP_003531556.1| interacted with gi|571,468,907|ref.|XP_006584499.1| (up), gi|351,723,797|ref.|NP_001234988.1| (up), gi|356,572,846|ref.|XP_003554576.1| (up), gi|356,556,926|ref.|XP_003546771.1| (up), gi|356,548,210|ref.|XP_003542496.1| (up) and gi|363,808,142|ref.|NP_001242479.1| (down); gi|356,563,232|ref.|XP_003549868.1| interacted with gi|356,544,142|ref.|XP_003540514.1| (up), and gi|351,727,873|ref.|NP_001235640.1| interacted with gi|356,500,108|ref.|XP_003518876.1| (up). The interaction network of the identified part of the DEPs in Wan82–178 (HS) was divided into five levels (Fig. 10b), namely gi|356,525,894|ref.|XP_003531556.1| interacted with gi|351,723,797|ref.|NP_001234988.1 | (up), gi|351,723,535|ref.|NP_001238050.1| (down), gi|356,507,166|ref.|XP_003522341.1| (down) and gi|356,572,874|ref.|XP_003554590.1| (down), gi|356,507,815|ref.|XP_003522659.1| and gi|356,517,518|ref.|XP_003527434.1| interacted with gi|356,512,203|ref.|XP_003524810.1| (down), gi|356,531,794|ref.|XP_003534461.1| interacted with gi|356,513,927|ref.|XP_003525659.1| (down), gi|356,568,923|ref.|XP_003552657.1| interacted with gi|356,568,923|ref.|XP_003552657.1| (down), and gi|356,501,755|ref.|XP_003519689.1| interacted with gi|571,450,890|ref.|XP_003523016.2| (up). Before *Lamprosema indicate* feeding, the interaction network of part of the DEPs in the insect susceptible material (HS0) was divided into four levels when compared to insect resistant material (HR0) (Fig. 10c), namely gi|356,525,894|ref.|XP_003531556.1|, gi|356,540,213|ref.|XP_003538584.1| and gi|356,501,755|ref.|XP_003519689.1| interacted with gi|356,548,210|ref.|XP_003542496.1| (up), gi|356,498,779|ref.|XP_003518226.1| (up), gi|356,556,926|ref.|XP_003546771.1| (up) and gi|356,516,623|ref.|XP_003526993.1| (down), respectively; gi|356,501,324|ref.|XP_003519475.1| interacted with gi|571,450,890|ref.|XP_003523016.2| (down), gi|356,507,815|ref.|XP_003522659.1| interacted with gi|571,488,294|ref.|XP_006590896.1| (down), and gi|356,512,636|ref.|XP_003525024.1| interacted with gi|356,530,364|ref.|XP_003533752.1| (down). After the interaction of up and down regulation, the expression levels of gi|356,525,894|ref.|XP_003531556.1|, gi|356,540,213|ref.|XP_003538584.1| and gi|356,501,755|ref.|XP_003519689.1| were unchanged. It was speculated that this might act as a bridge or signal transduction in the middle part of the regulatory network. After the up or down regulation of single proteins, the other proteins, such as the expression levels of gi|356,563,232|ref.|XP_003549868.1|, gi|351,727,873|ref.|NP_001235640.1|, gi|356,507,815|ref.|XP_003522659.1|, gi|356,517,518|ref.|XP_003527434.1|, etc. were unchanged. It was presumed that this was a result of invalid control or regulated by other factors, thus there was no significant change in expression. The results of this study showed that the difference expressions of related proteins in the soybean stimulated the regulation of *Lamprosema indicate* feeding stress response, causing the plant body endocrine to create more antigen proteins, so as to resist the harm caused by the *Lamprosema indicate*.

### Validation of iTRAQ data for selected candidate proteins by multiple reaction monitoring (MRM)

**Fig. 11:**
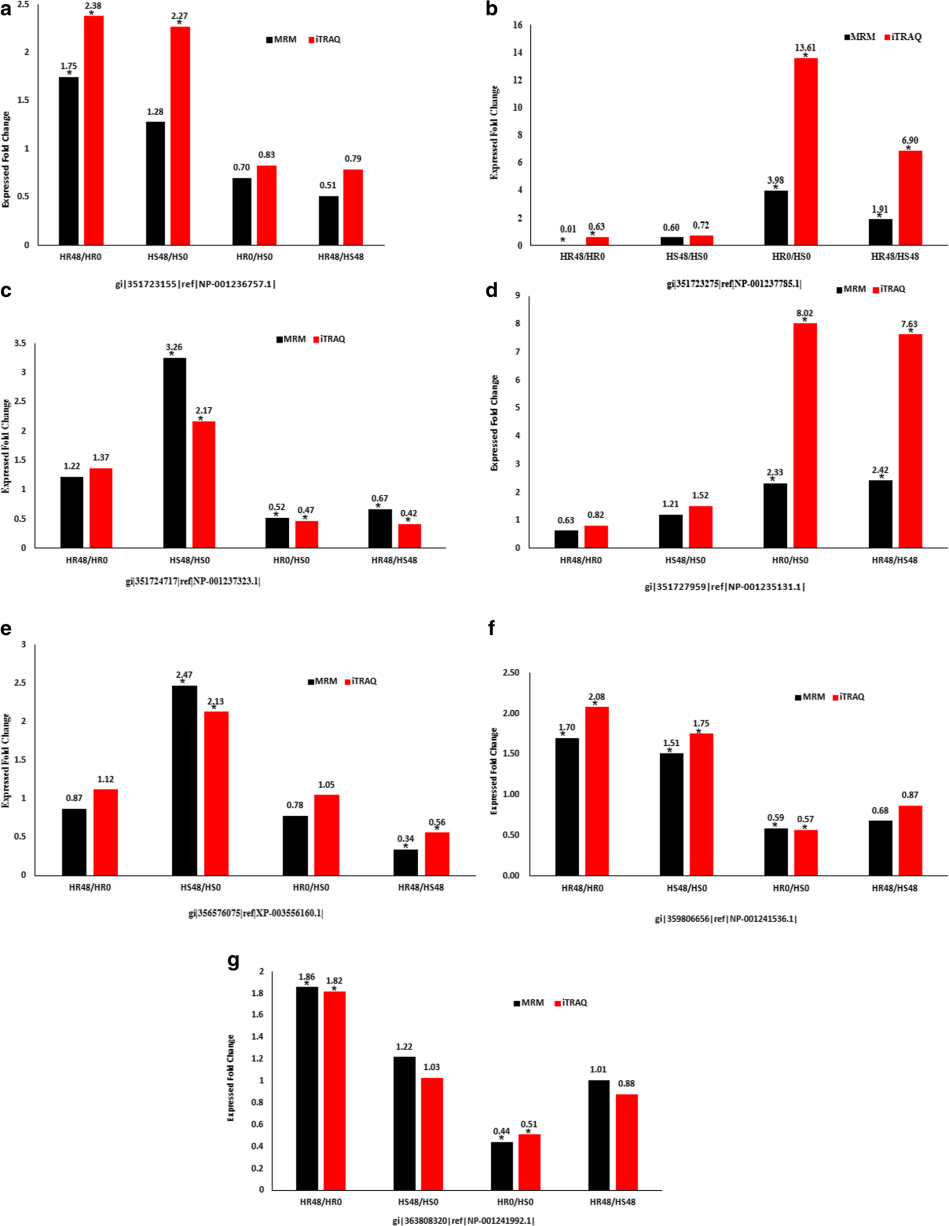
Relative expression leves of selected proteins measured by iTRAQ and MRM in the HR48/HR0, HS48/HS0, HR0/HS0 and HR48/HS48. **a**. gi|351,723,155|ref.|NP-001236757.1|, **b**. gi|351,723,275|ref.|NP-001237785.1|, **c**. gi|351,724,717|ref.|NP-001237323.1|, **d**. gi|351,727,959|ref.|NP-001235131.1|, **e**. gi|356,576,075|ref.|XP-003556160.1|, **f**. gi|359,806,656|ref.|NP-001241536.1|, **g**. gi|363,808,320|ref.|NP-001241992.1|. Note: “*” means DEPs. HR represents the highly resistant line of the Gantai-2-2; HS represents the highly susceptible line of the Wan 82–178; and 0, 48 indicate that the soybean samples were treated with *Lamprosema indicate* at 0 and 48 h, respectively

The reliability of the iTRAQ results was verified by MRM. In accordance with the reported data results, as well as the different expression proteins analyses, 11 candidate DEPs which may have been related to resistance to *Lamprosema indicate* were selected to establish an MRM method. Skyline software was used to select peptides of the target proteins with a MS/MS spectral library (cut-off score: 0.95) which were generated on a TripleTOF5600 (AB SCIEX) search using Mascot (Matrix Science, UK) against with a *Glycine_max* database. Among 11 target proteins, 7 proteins have MS/MS spectrum (s) and unique peptide (s). Therefore, the MRM detection and analysis were performed for these 7 DEPs only (Additional file 1: Table S3 and Additional file 2: Figure S1, S2, S3). The results showed that the expression levels of the 7 DEPs in the different comparison programs were basically the same as in the iTRAQ expression patterns (Fig. 11). The difference between the levels of expression may have been due to the different detection methods. Therefore, the MRM analysis confirmed that the iTRAQ results were reliable.

## Discussion

The different resistance soybeans formed self-defense proteins in order to avoid the insect herbivory attacks before *Lamprosema indicate* feeding stress. Also, after feeding some defense related proteins expressions were induced, which regulated the plants’ metabolic balance in order to achieve insect resistant reactions. For the purpose of more thoroughly understanding the protein expression levels in the soybean after the *Lamprosema indicate* feeding for 0 h and 48 h, a differential proteomic analysis was conducted for the leaves of the highly resistant line (Gantai-2-2) and the highly susceptible line (Wan 82–178) using iTRAQ. A total of 82 DEPs were identified according to the standard threshold. Then, based the biological information analyses of these DEPs, combined with the previously reported data, some of the DEPs were determined to be related to the *Lamprosema indicate*.

### DEPs associated with active oxygen scavenging

Plants may be subjected to drought, high and low temperature, salt, plant disease and pest stress during their entire growth process. All of these can lead to the plants’ cells producing large amounts of reactive oxygen species (ROS) [18]. ROS are important signal molecules in plants, but can also cause plant toxicity. Plants have formed a set of regulations and detoxification mechanisms, along with enzymatic and non-enzymatic antioxidant protection systems, to remove the ROS [19, 20].

Peroxidase (POD) is an important defense enzyme system in plants. It has the ability to catalyze the decomposition of H_2_O_2_ to remove the ROS [21–24], which can be induced by insects in wheat [25, 26], tomato [27], sorghum [28], cucumber [29], rice [4, 6], and other crops. In this study, the peroxidase 12-like in the Wan 82–178 was up-regulated compared 48 h with 0 h. It was speculated that this protein in the highly susceptible soybean had the ability to reduce the damage of the ROS which was caused by the invasion of the pest.

Ascorbate peroxidase (APX) is a key enzyme in the H_2_O_2_ detoxification system, can catalyze cycle it plays the key role of catalyzing the AsA-GSH cycle [30, 31]. The APX isozymes in higher plants are divided into cytosolic APX (cAPX), and chloroplasts APX (chlAPX), in which the cAPX are the main enzymes for responses to harsh environments [32]. Caldwell et al. [33] found that the transcription, translation, and post translation regulations of the cAPX in soybean could enhance its ability to resist environmental stress. Yoshimura et al. [34] determined that the absence of cAPX1 caused a breakdown of the chloroplast H_2_O_2_ clearance system. In this study, it was found that the expression level of the cAPX1 in the Gantai-2-2 was always higher than that in the Wan 82–178 at 0 h and 48 h. These results indicated that expression of cAPX1 can protect the important regions of the cells from oxidative stress, and strictly control the intracellular H_2_O_2_ levels in order to resist pest invasions [31].

Superoxide dismutase (SOD) in plants is a key enzyme in the removal of ROS. It is an important protective enzyme in plants’ cell defense systems, and is closely related to the plants’ resistances [35]. Depending on the combination of metal ions, SOD can be divided into three types: Cu/Zn-SOD, Mn-SOD, and Fe-SOD [36]. Previous studies have confirmed that salt stress [37], cucumber mosaic virus (CMV) [38] and plum pox virus (PPV) [39] can all induce the expression of SOD in plants. Also, oxidative stress, hormones, and salt stress adversity can induce the Fe-SOD expression in barley [40], tobacco [41, 42], soybean [43], and other crops. The two Fe-SOD proteins in the Gantai-2-2 were up-regulated compared 48 h with 0 h, and illustrated that the highly resistance materials under the *Lamprosema indicate* stress can decrease the damages of the ROS, then resist pests. Also, the expression level of two Fe-SOD proteins in the Wan 82–178 were always higher than that in the Gantai-2-2 at 0 h and 48 h. Therefore, a high expression of Fe-SOD in the highly susceptible soybean was determined to be a defense against pest invasion.

### DEPs related to other secondary metabolite biosynthesis

Cytochrome P450 (CYP) is a class of heme containing oxidoreductases. It can catalyze some substances with defensive functions, such as isoflavones, alkaloids, terpenoids, and so on [44]. It plays an important role in the defense against pests, as well as environmental stresses [45]. The products were catalyzed by CYP79A and CYP71E1 in a sorghum containing cyanide, which is toxic to insect pests [46]. The phytoalexins in *Arabidopsis* were catalyzed by CYP71B15, which can resist pathogen hazards [47]. Transgenic maize expressing cytochrome P450 genes were able to avoid herbicide toxicity [48]. BPHs can induce the cytochrome P450 expression in rice [4]. In this study, a cytochrome P450 82A4-like was up-regulated in the Wan 82–178 compared 48 h with 0 h. And it was found to be always higher in the Gantai-2-2 than in the Wan 82–178 at 0 h and 48 h. Therefore, it speculated that the cytochrome P450 82A4-like catalyzed the synthesis of the substances which were defending against the pest, and thereby caused harm to the insects or stopped the pests’ feeding.

One of the key enzymes in the ethylene biosynthesis of plants is 1-aminocyclopropane-1- carboxylate oxidase (ACO). The amount of ethylene production is mainly determined by the activity of the ACO [49]. The ACO is a multigene family encoding protein, which is regulated by the feedback regulation of the ethylene signaling [50]. Ethylene, as a plant endogenous hormone, regulates the growth and development of plants, defense responses, and the synthesis of secondary metabolites [51]. It was found that the ACO would be induced when peach was affected by wounding [52]. The NtACO1, NtACO2 and NtACO3 in tobacco were induced during salt stress [53, 54]. Also, the ACO in a wild type of *Arabidopsis* was enhanced after being treated for salt resistance [55]. In this study, the ACO in the Wan 82–178 was up-regulated compared 48 h with 0 h. It was hypothesized that the pest stress inducted the ACO expression, and produced a large amount of ethylene in order to improve the resistance abilities to the *Lamprosema indicate*.

OAS-TL is a key enzyme in the synthesis of Cys, and it has been found to be closely related to the secondary metabolic synthesis of some plants [56]. Previous studies have shown that the OAS-TL isoenzymes in watermelon [57], *Leucaena leucocephala* [58] and *Quisqulis indica* [59] can synthase toxic or anthelmintic beta heterocyclic rings, which replaced alanine and resisted pests. In this study, the expression level of the *GmOASTL4* in Gantai-2-2 was found to always be higher than the Wan 82–178 at 0 h and 48 h. These results suggested that it may synthase some of the secondary products resistance to *Lamprosema indicate* feeding, or beta site heterocyclic substituted alanine and other substances. Therefore, it plays a key role in the defense against *Lamprosema indicate*.

### DEPs associated with carbohydrate metabolism

Glutamine synthetase (GS) is the key enzyme of the nitrogen metabolisms in higher plants. It catalyzes the ammonium salt and glutamate to generate glutamine, providing needed nitrogen compounds for proline synthesis, which affects plant growth and development [60]. GS is divided into two categories, cytoplasmic or cytosolic types of GS (GS1), and plastid or chloroplast types of GS (GS2), in which the GS1 mainly produces glutamine to complete the transport of nitrogen between cells [61]. Previous studies have shown that the GS1 isozyme is associated with plant disease and stress resistances. The expression of the GS1 isozyme was enhanced when the following plants suffered pathogen infections, drought, and other stresses: tomato [62, 63], tobacco [64], potato [65] and soybean [66]. In this study, the GS1 isoform X1 was all up-regulated in the Gantai-2-2 and Wan 82–178, comparing 48 h with 0 h, which may have facilitated proline accumulation, in order to improve the osmotic adjustment abilities in the soybean leaves, as well as to activate the oxygen scavenging capacities against the *Lamprosema indicate* feeding.

Enolase is a key enzyme of the glycolytic pathway, and has the ability to catalyze the dehydration of phosphoglyceric acid to form phosphoenolpyruvic acid [67, 68]. It has been reported that enolase is involved in stress responses, which can be induced by pest invasions, hypoxia, and cold stress in rice [69, 70], maize [71], cotton [72], soybean [73] and other plants. In this study, the enolase was up-regulated in the Wan 82–178, comparing 48 h with 0 h. It was speculated that the expression changes of the enolase may promote the light respiration in the plants, thereby maintaining a dynamic equilibrium in order to resist pests [72].

### DEPs associated with jasmonic acid (JA) signaling pathways

When plants are subjected to pest feeding or stress, they will be induced to produce jasmonic acid (JA)-mediated signaling pathways. Ellis et al. [74] found that aphid populations’ growth decreased significantly in the *Arabidopsis* mutant *cev1* when the JA signaling pathway was activated. These results indicated that JA limits the damage to plants caused by aphids. Lipoxygenase (LOX) is the key enzyme in the synthesis of JA [75], and also is an important signal factor in plants’ defense pathways [76]. When plants suffer pest infestations, pathogen infections, high temperature, and other stresses, the expression of single or multiple LOX genes can be induced [77, 78]. These have been induced by pests, water logging, hypoxia, or disease adversity stresses in the following plants: potato [79], tomato [80, 81], *Phaseolus vulgaris* [82], soybean [73, 83], *Arabidopsis thaliana* [84–86], and *Citrus sinensis* [87], as well as other plants. Constabel found that the LOX activity increased rapidly, can led to a slowdown of the development speed of *Spodoptera exigua* [88]. Alpha-dioxygenase (α-DOX) is also able to catalyze the oxidation of fatty acids. The product of 2-hydrogen peroxide fatty acids is a new type of oxidation fatty acid, and is also an enzyme in the JA pathway [4]. Some studies have indicated that α-DOX is also related to plants’ resistances to pathogen invasions and insect attacks. α-DOX1 or α-DOX2 have been induced by plant diseases, insect pests, or adversity stresses in *Arabidopsis thaliana* [89], tobacco [90], tomato [91] and rice [4, 92]. In this study, seven LOX and one α-DOX1 were all up-regulated in the Gantai-2-2 and Wan 82–178, comparing 48 h with 0 h. It was speculated that the linolenic acid in the soybean leaves was released from the membrane lipids during the damage caused by the pest invasion, and the JA and its derivatives were finally formed under the action of the LOX and α-DOX1 [93]. JA is a plant’s signal molecule caused by injury, herbivores, and pathogens [94], and binding to a receptor can induce the expression of defense enzymes or proteins [95]. It was presumed the JA signaling pathway was involved in the responses to the *Lamprosema indicate* defense in the plants.

### DEPs related to genetic information processing

Many defense related proteins are produced when plants are being damaged by insects, many of which are synthesized by the endoplasmic reticulum, and transported by the Golgi apparatus to various parts of the cells [96]. In this study, protein disulfide-isomerase-like (PDI), PDI A6, and calreticulin-3-like (CRT-3) were all involved in the process of protein synthesis in the endoplasmic reticulum. The PDI which resides in the endoplasmic reticulum could catalyze the protein disulfide oxidation, reduction, and isomerization through the endoplasmic reticulum signal peptide [97]. Many previous studies have shown that PDI is involved in stress responses, such as drought, cold, and salt stresses, which have induced PDI expressions in corn [97], rice [98] and soybean [99]. CRT is a multifunctional Ca^2+^ binding protein, and is mainly attached to the endoplasmic reticulum. CRT is involved in plant stress responses under biotic and abiotic stresses [100]. The CRT was found to be up-regulated by mosaic virus infections or waterlogging stresses in soybean [101, 102]. In this study, the PDI, PDI A6 and CRT-3 in the Wan 82–178 were all up-regulated, comparing 48 h with 0 h. Therefore, it was speculated that the highly susceptible material activated the basic defense mechanisms after the *Lamprosema indicate* feeding. However, other resistance mechanisms existed in the highly resistant material.

### Anti digestion proteins

Lectins have at least one non-catalytic domain specific reversible binding to monosaccharide or oligosaccharide. When lectin is ingested by insects to induce local or systemic toxic effects, it may result in antifeedant, insect growth arrest, and even death [103]. Previous studies have shown that the survival and reproductive rates were greatly reduced in *Heliothis virescens* [104], *Myzus persicae* [105], *Lacanobia oleracea* [106], and other insects, which fed on transgenic plants expressing the lectin. Transgenic tobacco which expressed soybean lectin enhanced its resistance capacity to pathogens and pests [107]. In this study, the lectin precursor was all up-regulated in the Gantai-2-2 and Wan 82–178, comparing 48 h with 0 h. Presumably, the lectin precursor was released from soybean victims’ cells, and then combined to the chitin of the insects’ peritrophic membranes, and sugar-based compounds and glycosylation of digestive enzymes in epithelial cells of the digestive tract, thereby affecting the normal absorption of nutrients, disorders in the digestive tract were also induced, and bacterial multiplication in the digestive tract was promoted, causing insect growth inhibition or death, which achieved the defensive purpose of killing the pests [108].

Trypsin inhibitors can significantly inhibit the protease activities in insects’ guts, and hinder the insects’ digested protein. They have especially strong inhibitory effects on lepidopteran insects [109–111]. The insects which have fed on trypsin inhibitors and have suffered from malnutrition and even increased mortality include *Tribolium castaneum* [112], *Spodopera exigua* [113], *Heliothis zea* [113], *Callosobruchus maculates* [114], *Apis mellifera* [115], and so on. In this study, trypsin inhibitor, trypsin inhibitor A-like and kunitz trypsin protease inhibitor-like precursors, were all up-regulated in the Gantai-2-2 and Wan 82–178, comparing 48 h with 0 h, which may have caused the proteolytic enzyme activities in the insects’ guts to decrease significantly, as well as disturbing the normal metabolisms of the insects.

### DEPs associated with resistance to *Lamprosema indicate*

PR-5 proteins are a class of the PRs protein family, and play a very important role in plants’ resistances to adversity stresses [116]. Previous study results have shown that the transgenic tomato expressing PR5 genes could increase disease resistance [117], and salt stress or disease induced the expression of the PR5 protein in tomato [118], wheat [119], and other plants. In this study, the PR-5b protein precursor in the Wan 82–178 was up-regulated, comparing 48 h with 0 h. Due to the multiple disulfide bonds of protein PR-5b maintaining the equilibrium distribution in the protein structure domain, the other proteins were protected [120, 121], or the PR-5 proteins increased the resistance through the accumulation of proline in the plants [122]. The soybean stress-induced protein SAM22 was expressed by the induction of wounding, or by the transpiration-mediated uptake of salicylic acid, methyl viologen, fungal elicitor, hydrogen peroxide, or sodium phosphate, as well as other stresses [123]. The expression of the stress-induced protein SAM22 in the soybean plants was up-regulated by waterlogging stress, and the expression was further strengthened when it was inoculated with *B. japonicum* [124]. In this study, the stress-induced protein SAM22-Iike was all up-regulated in the Gantai-2-2 and Wan 82–178, comparing 48 h with 0 h. These results indicated that stress defense system, as well as the relevant stress-induced protein SAM22, were involved in the defense responses.

## Conclusions

This study analyzed the differentially expressed proteomic for soybean, the resistant and susceptible varietal and varieties after *Lamprosema indicate* feeding and non-feeding using iTRAQ technology, as well as the screening the DEPs which were resistant to *Lamprosema indicate*. The results showed that 28 DEPs were identified in the Gantai-2-2 when compared to the Wan 82–178 at the *Lamprosema indicate* non-feeding. A comparison was made of the *Lamprosema indicate* feeding at 48 h and 0 h, 31 DEPs were identified in the Gantai-2-2 and 53 DEPs were identified in the Wan 82–178. All of the identified DEPs were further divided into three types: non-*Lamprosema indicate*-induced, both genotypes of insect-induced differential proteins, and *Lamprosema indicate*-induced differential proteins. Different resistance soybeans may use different defense strategies in response to pests, in which highly susceptible soybeans boost more of the proteins involved in the responses to pest stresses. Based on the results of the differential proteomic analyses, along with the relevant previously released data, it was suggested that the soybean defended or resisted the *Lamprosema indicate* damage by the induction of a synthesis of anti-digestive proteins which inhibit the growth and development of insects, reactive oxygen species scavenging, signaling pathways, and secondary metabolites synthesis, and so on.

## Methods

### Plant materials

In this study, the tested soybean varieties were Gantai-2-2 (highly resistant line, HR) [10] and Wan 82–178 (highly susceptible line, HS) [10]. The samples were planted in an experimental field within insect screen rooms at the Guangxi Academy of Agricultural Sciences on March, 2015. They were sown in a three-row division. During the entire growth period of soybeans, the spraying pesticides and fertilizers was not used. When the plants grew to a level of ten compound leaves, the seventh compound leaves on the left side were collected before the infestation. There were a total of five plants for each sample, as well as two repetitions for each sample. Then, each of the samples was simultaneously artificially infested with five four-year-old *Lamprosema indicatas* of on the right side of the seventh countdown leaves. The samples were collected at 48 h after the insect feeding, quickly frozen using liquid nitrogen, and refrigerated at −80 °C for storage purposes.

### Protein preparation

The samples were placed in a mortar, then 10% PVPP was added, and the samples were ground into a powder in liquid nitrogen. Then, 2 g of the ground powder was taken and added to Lysis buffer (7 M Urea, 2 M thiourea, 4% CHAPS, 40 mM Tris-HCl, pH 8.5) containing 1 mM PMSF and 2 mM EDTA. The samples were placed on ice for 5 min, after which 10 mM DTT was added. The samples were sonicated at 200 W for 5 min, then centrifuged at 4 °C and 15,000×*g* for 15 min. Next, the supernatant was transferred to another tube, mixed thoroughly with 5*×* volume of cold acetone containing 10% (*v*/v) TCA (trichloroacetic acid), and added 10 mM DTT, and finally precipitated in the solution at −20 °C overnight. After centrifugation at 4 °C and 25,000×*g* for 15 min, the supernatant was discarded. The sediment was then collected, 1 mL cold acetone and 10 mM DTT were added, followed by mashing and precipitation, then the solution was placed at −20 °C for 30 min. After centrifugation at 4 °C and 25,000×*g* for 15 min, the supernatant was discarded. The precipitate was washed with cold acetone three times. The pellet was air dried and dissolved in Lysis buffer (7 M Urea, 2 M thiourea, 4% CHAPS, 40 mM Tris-HCl, pH 8.5) containing 1 mM PMSF and 2 mM EDTA was added, after 5 min added 10 mM DTT. The suspension was sonicated at 200 W for 15 min, and centrifuged at 4 °C and 25,000×*g* for 15 min. The supernatant was transferred to another tube, 10 mM DTT was added, and the solution was incubated at 56 °C for 1 h in order to open the disulfide bond. Then, 55 mM IAM was added to the block cysteine, which was incubated for 45 min in the darkroom. The supernatant was mixed with 55*×* volume of cold acetone at −20 °C for 2 h. After centrifugation at 4 °C and 25,000×*g* for 20 min, the supernatant was discarded. The pellet was air-dried for 15 min, dissolved in 200 μL 0.5 M TEAB (Applied Biosystems, Milan, Italy), and sonicated at 200 W for 15 min. Finally, the samples were centrifuged at 4 °C and 25,000×*g* for 20 min, and the supernatant was obtained and used for the quantitative analysis.

### Protein quantification and concentration detection

The protein concentration was determined using a Bradford assay, with a bovine serum albumin (BSA) concentration as the standard [125]. The protein quality and concentration were detected using SDS-PAGE. The protein samples obtained were stored at −80 °C.

### Protein digestion

Total protein (100 μg) was removed from each sample solution and digested with Trypsin Gold (Promega, Madison, WI, USA) at a protein: enzyme ratio of 20:1 at 37 °C for 4 h. Trypsin was once again added according to the above ratio, and digested at 37 °C for 8 h. Following the trypsin digestion, the peptide was dried using a vacuum centrifugal pump.

### iTRAQ labeling and SCX fractionation

The iTRAQ test and its results were analyzed by the Shenzhen Institute of Gene Research (BGI, Shenzhen, Guangdong Province, China). Peptides were reconstituted in 0.5 M TEAB and labeled using 8-plex iTRAQ reagent (Applied Biosystems, CA, USA) according to the manufacturer’s introductions. Each unit of the iTRAQ reagent was thawed and reconstituted in 24 μL isopropanol. The Gantai-2-2 was fed on by the *Lamprosema indicata* at 0 h using 113 and 115 tags, at 48 h using 117 and 119 tags; the Wan 82–178 at 0 h using 114 and 116 tags, at 48 h using 118 and 121 tags. Then, each group of peptide segments was labeled with different iTRAQ tags, and cultured at room temperature for 2 h. Then all of the labeled groups were mixed and dried by vacuum pumping.

The sample was separated using a Shimadzu LC-20AB HPLC Pump system (Shimadzu, Kyoto, Japan), and the separation column was an Ultremex SCX (4.6 × 250 mm, Phenomenex, CA, USA). All of the labeled peptides were dissolved using 4 mL buffer A (25 mM NaH_2_PO_4_ in 25% ACN, pH 2.7) and loaded onto a 4.6 × 250 mm Ultremex SCX column 5 μm particles. The peptides were eluted at a flow rate of 1 mL/min with a gradient elution of buffer A for 10 min, 5–60% buffer B (25 mM NaH_2_PO_4_, 1 M KCl in 25% ACN, pH 2.7) for 27 min, 60–100% buffer B for 1 min, The system was then maintained at 100% buffer B for 1 min before equilibrating with buffer A for 10 min prior to the next injection. The entire elution process was monitored at 214 nm absorbance, and fraction were collected every 1 min. The eluted peptides were pooled into 20 fractions. Then, the salt was removed from each fraction using Strata X C18 (Phenomenex, CA, USA), and lyophilized for storage purposes.

### LC- ESI- MS/MS analysis based on TOF triple 5600

Each fraction was dissolved with buffer A (5% ACN, 0.1% FA) and centrifuged at 20,000×*g* for 10 min, the final concentration of 0.5 μg/μL on average. 10 μL supernatant was loaded on a LC-20 AD nanoHPLC (Shimadzu, Kyoto, Japan) by the auto-sampler onto a 2 cm C18 trap column. Then, the peptides were eluted onto a 10 cm C18 column (inner diameter 75 μm) packed in-house. The samples were loaded into the trap column at a flow rate of 8 μL/min for 4 min, then the 35 min gradient was run at 300 nL/min starting from 5% to 35% buffer B (95% ACN, 0.1% FA), which was then increased to 60% within the next 5 min. Then, buffer B increased to 80% within 2 min, and held for 2 min. Finally, it was restored to 5% within 1 min, and the balance was maintained under this condition for 10 min.

Following the liquid phase separation, the peptides were input into a series ESI mass spectrometer, which was a triple TOF 5600 type (AB SCIEX, Concord, MA, USA). The ion source was a Nanospray III (AB SCIEX, Concord, MA, USA), and the emitter was a spray needle, which was made from quartz material (New Objectives, Woburn, MA, USA). During the data acquisition, the machine parameters were set as follows: the spray voltage of the ion source was 2.5 kV; nitrogen pressure was 30 psi (14.5 psi = 1 bar); the spray pressure was 15 psi; the temperature of the spray interface was 150 °C; the scanning mode was reflection mode, and the resolution was not less than 30,000; 250 ms were accumulated in the first MS, and only scan was charged from 2+ to 5+; the first 30 scanned strengths were more than 120 cps, with 3.3 s per cycle; the second quadrupole (Q2) transmission set was at the rate of 100% at 100 Da; the frequency rate of the electric pulse RF was 11 kHz; the detection frequency rate of the detector was 40 GHz; and the particle signals of each scan with four channels were recorded four times, then merged into data. For the iTRAQ project, the energy of the ion fragmentation was set to 35 ± 5 eV, and the parent ion dynamic exclusion set was set to half of the peak time (approximately 15 s). Also, the fragmentation of the same parent ion was no more than twice.

### iTRAQ data analysis

The original document of the mass spectrometer was identified in order to obtain the peak list. The original file of the mass spectra which was obtained from Orbitrap was transferred into MGF file format using Proteome Discoverer 2.2 software (PD2.2, Fisher Scientific Thermo, Waltham, MA, USA). Proteins identification were performed by using Mascot search engine (Matrix Science, London, UK; version2.3.02) against the NCBI *Glycine_max* database contenting 66,116 sequence (ftp.ncbi.nih.gov/genomes/Glycine_max/protein[Organism:noexp]).

For the protein identification, a mass tolerance of the ppm was permitted to form 20 intact peptide masses, and 0.05 Da for the fragmented ions, with allowances made for one missed cleavage in the trypsin digestion. The Gln- > pyro-Glu (N-term Q), oxidation (M), and deamidated (NQ) were the potential variable modifications, and carbamidomethyl (C), iTRAQ8plex (N-term), and iTRAQ8plex (K) were the fixed modifications. The charge states of the peptides were set to +2 and +3. Specifically, an automatic decoy database search was performed in Mascot by choosing a decoy checkbox in which a random sequence of databases was generated and tested for raw spectra, along with the actual database. In order to reduce the probability of false peptide identification, only peptides with significance scores (≥ 20) at the 99% confidence interval by a Mascot CE probability analysis greater than “identity” were counted as identified.

Student’s t-test was used for significance evaluation when only two groups were compared in each repetition. The minimum requirements for DEPs were at least two matched unique peptides and a significant change (*P* ≤ 0.05 and ≥1.5-fold or ≤0.67-fold change) in protein quantities between the stress-treated samples and control samples in at least one repetition, with the other repetition displaying a similar trend.

### Bioinformatic analyses

Clustering analysis is a method which is widely used in pattern recognition and data mining, and is an effective method based on data knowledge discovery. In this study, Cluster 3.0 software (Stanford University, USA) was used for the hierarchy clustering (HC) analysis of the quantitative data of the DEPs in four comparative groups [126]. The calculation of the Euclidean distance between groups of data was performed by using the standard change of data value, and the identified protein and experimental conditions were conducted simultaneously by grade cluster analysis, while the results of clustering analysis were shown by Java Treeview [127]. The Euclidean distance was closer, which indicated that the properties of the two sets of data were closer, while the Euclidean distance was farther, thus indicating that the association was farther.

Gene Ontology (GO) is an international standardization system of gene function classification. It provides a dynamic updating of the standard vocabulary table (controlled vocabulary) in order to fully describe the properties of organisms’ genes and gene products. The GO contains three ontologies, which describe the genes’ molecular functions, cellular components, and biological processes [128]. A Cluster of Orthologous Groups of proteins (COG) is a database for the proteins of orthologous classification, and each protein in a COG is assumed to have been derived from an ancestral protein. The identified protein will blast with the COG database to predict these proteins’ functions, and to create statistics of the functions’ classifications [129]. In vivo, the coordination of the different proteins exercise their biological behaviors, and this is helpful to the further understanding of the biological functions based on pathway analysis. The Kyoto Encyclopedia of Genes and Genomes (KEGG) is a major public database related to pathways [130]. It has the ability to identify the majority of the important proteins involved in biologic metabolic and signal transduction pathways.

In the present study, the functional annotations of the DEPs were performed by utilizing the Blast2go_v2.5 program against the NCBInr and UniProt/SwissProt database. A functional classification of the proteins using COG enrichment (http://www.ncbi.nlm.nih.gov/COG/) was performed on all of the identified DEPs using Blastx/Blastp 2.2.24 software. Then, all of the identified DEPs were mapped to a pathway in the KEGG database (http://www.genome.jp/kegg/pathway.html) using Blastx/Blastp 2.2.24. A *p*–value was used as the threshold ≤0.05, in order to judge the significance of the GO, COG and KEGG pathway enrichment analyses.

The analysis of the protein interaction network was constructed by String (http://string-db.org/) and Cytoscape3.0 (http://www.cytoscape.org/). The predicted protein sequence file (/STRING/homo_net.fa) was submitted to the String server, then the soybean information was selected and input into the soybean database. Next, soybean was selected as the species, “Swiss-port” as the database, and biological pathway as the GO process, then these were submitted for prediction. The protein interaction data were visualized by the network visualization software program Cytoscape3.0.

### Analysis of the MRM

MRM is a type of mass spectrometry technology, which is applied to the quantitative analysis of target proteins based on the known or presumed information of the protein, for the purpose of obtaining the data and collecting the spectrum signals [131]. MRM is suitable for the detection of the reliability of iTRAQ mass spectrometry at a protein level. Samples were reconstituted in 0.5 M TEAB, and digested as described elsewhere and spiked with 40 fmol of β-galactosidase for data normalization. MRM analysis was performed on a QTRAP5500 mass spectrometer (AB SCIEX, Foster City, CA) equipped with Waters nano Acquity Ultra Performance LC system. The Mobile phase consisted of solvent A, 0.1% aqueous formic acid and solvent B, 98% acetonitrile with 0.1% formic acid. Peptides were separated on a BEH130 C18 column (0.075 × 200 mm column, 1.7 μm; Waters) at 300 nL/min, and eluted with a gradient of 2–40% solvent B for 30 min, 40–60% solvent B for 3 min, and followed by 2 min linear gradient to 80% solvent B and maintenance at 80% for 5 min. For the QTRAP5500 mass spectrometer, spray voltage of 2100 V, nebulizer gas of 20 p.s.i., and a dwell time of 10 ms were used. Multiple MRM transitions were monitored using unit resolution in both Q1 and Q3 quadrupoles to maximize specificity.

A spectral library of MS/MS data was searched using Mascot v2.3 (Matrix Science, UK) against with a *Glycine_max* database. The data file was imported into Skykin software (1.2.0.3425 Skyline) where a library was built [132]. The peptides was selected for MRM method development according to the following criteria: the peptides with unique sequence in the database; a maximum m/z of peptide <1250 (limination of Quandrupole scan), with a peptide length range 5–40 aa; without methionine in peptides; with carbamidomethyl on cysteine and without variable modification in peptides; and no missed cleavage of trypsin. The predicted retention time of targeted peptides was observed with a iRT strategy [133]. A pooled peptides digested as described was performed preliminary SRM assays used to determine where these proteins were detected.

MRM method of a given protein was successfully developed only if the protein had at least one unique peptide which was identified with MS/MS spectral library (cut-off score > 0.95), had >5 fragment ions with the same elution profile and in the same ratios as the spectral library, and had an accurate retention time (less than ±2 min deviation against to predicted retention time). A significant change (*P* ≤ 0.05 and ≥1.5-fold or ≤0.67-fold change) in protein quantities between the stress-treated samples and control samples in at least one repetition, with the other repetition displaying a similar trend.

## Additional files

Additional file 1: Table S1. Overview of protein identification results of soybean [*Glycine max* (L.) Merr.] leaves proteins by iTRAQ-LC/MSMS method. **Table S2.** Raw iTRAQ data for protein identification and quantitation. **Table S3.** Detail information of each transition. (ZIP 2100 kb)

Additional file 2: Figure S1. Correlation between predicted retention time and observed retention time. **Figure S2.**-A MS/MS spectrum of a given peptide of gi351723671refNP-001237543-1_WVAFVDNEIQK_MS2. **Figure S2.**-B Dot-product of a given peptide of gi351723671refNP-001237543-1_WVAFVDNEIQK_MS2. **Figure S2.**-C MRM chromatogram of a given peptide of gi351723671refNP-001237543-1_WVAFVDNEIQK_MS2. **Figure S3**-A MS/MS spectrum of a given peptide of gi351724717refNP-001237323-1_GLFEGGIHLPTDALSK_MS2. **Figure S3.**-B Dot-product of a given peptide of gi351724717refNP-001237323-1_GLFEGGIHLPTDALSK_MS2. **Figure S3.**-C MRM chromatogram of a given peptide of gi351724717refNP-001237323-1_ GLFEGGIHLPTDALSK_MS2. (ZIP 197 kb)

## Abbreviations

ACO: 1-Aminocyclopropane-1-carboxylate oxidase
APX: Ascorbate peroxidase
BPH: Brown planthopper
COG: Cluster of orthologous groups of proteins
CRT-3: Calreticulin-3-like
CYP: Cytochrome P450
DEPs: Differentially expressed proteins
GO: Gene ontology
GS: Glutamine synthetase
iTRAQ: isobaric tag for relative and absolute quantitation
JA: jasmonic acid
KEGG: Kyoto encyclopedia of genes and genomes
LOX: Lipoxygenase
MRM: Multiple reaction monitoring
PDI: Protein disulfide-isomerase-like
ROS: Reactive oxygen species
SOD: Superoxide dismutase
α-DOX: Alpha-dioxygenase
