# Supplementary figures and images for: Proteomic analysis by iTRAQ-MRM of soybean resistance to Lamprosema Indicate
Source: BMC Genomics. 2017 Jun 6;18:444. doi: 10.1186/s12864-017-3825-0 (PMC5461738; doi:10.1186/s12864-017-3825-0)

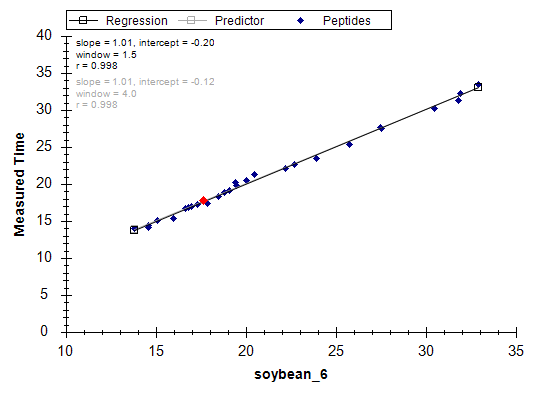

Supplement: Supplementary file 2 — Correlation between predicted retention time and observed retention time. Figure S2.-A MS/MS spectrum of a given peptide of gi351723671refNP-001237543-1_WVAFVDNEIQK_MS2. Figure S2.-B Dot-product of a given peptide of gi351723671refNP-001237543-1_WVAFVDNEIQK_MS2. Figure S2.-C MRM chromatogram of a given peptide of gi351723671refNP-001237543-1_WVAFVDNEIQK_MS2. Figure S3-A MS/MS spectrum of a given peptide of gi351724717refNP-001237323-1_GLFEGGIHLPTDALSK_MS2. Figure S3.-B Dot-product of a given peptide of gi351724717refNP-001237323-1_GLFEGGIHLPTDALSK_MS2. Figure S3.-C MRM chromatogram of a given peptide of gi351724717refNP-001237323-1_ GLFEGGIHLPTDALSK_MS2. (ZIP 197 kb) [file 12864_2017_3825_MOESM2_ESM.zip › Fig.S1..tif]

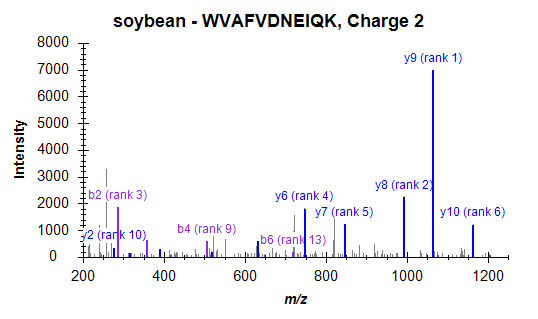

Supplement: Supplementary file 2 — Correlation between predicted retention time and observed retention time. Figure S2.-A MS/MS spectrum of a given peptide of gi351723671refNP-001237543-1_WVAFVDNEIQK_MS2. Figure S2.-B Dot-product of a given peptide of gi351723671refNP-001237543-1_WVAFVDNEIQK_MS2. Figure S2.-C MRM chromatogram of a given peptide of gi351723671refNP-001237543-1_WVAFVDNEIQK_MS2. Figure S3-A MS/MS spectrum of a given peptide of gi351724717refNP-001237323-1_GLFEGGIHLPTDALSK_MS2. Figure S3.-B Dot-product of a given peptide of gi351724717refNP-001237323-1_GLFEGGIHLPTDALSK_MS2. Figure S3.-C MRM chromatogram of a given peptide of gi351724717refNP-001237323-1_ GLFEGGIHLPTDALSK_MS2. (ZIP 197 kb) [file 12864_2017_3825_MOESM2_ESM.zip › Fig.S2-A.tif]

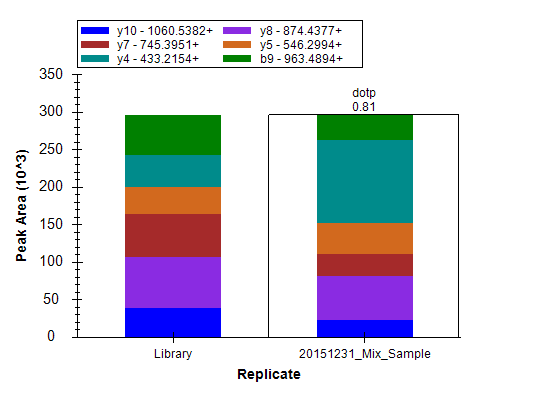

Supplement: Supplementary file 2 — Correlation between predicted retention time and observed retention time. Figure S2.-A MS/MS spectrum of a given peptide of gi351723671refNP-001237543-1_WVAFVDNEIQK_MS2. Figure S2.-B Dot-product of a given peptide of gi351723671refNP-001237543-1_WVAFVDNEIQK_MS2. Figure S2.-C MRM chromatogram of a given peptide of gi351723671refNP-001237543-1_WVAFVDNEIQK_MS2. Figure S3-A MS/MS spectrum of a given peptide of gi351724717refNP-001237323-1_GLFEGGIHLPTDALSK_MS2. Figure S3.-B Dot-product of a given peptide of gi351724717refNP-001237323-1_GLFEGGIHLPTDALSK_MS2. Figure S3.-C MRM chromatogram of a given peptide of gi351724717refNP-001237323-1_ GLFEGGIHLPTDALSK_MS2. (ZIP 197 kb) [file 12864_2017_3825_MOESM2_ESM.zip › Fig.S2-B.tif]

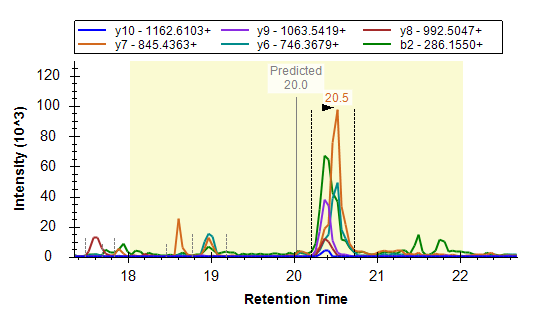

Supplement: Supplementary file 2 — Correlation between predicted retention time and observed retention time. Figure S2.-A MS/MS spectrum of a given peptide of gi351723671refNP-001237543-1_WVAFVDNEIQK_MS2. Figure S2.-B Dot-product of a given peptide of gi351723671refNP-001237543-1_WVAFVDNEIQK_MS2. Figure S2.-C MRM chromatogram of a given peptide of gi351723671refNP-001237543-1_WVAFVDNEIQK_MS2. Figure S3-A MS/MS spectrum of a given peptide of gi351724717refNP-001237323-1_GLFEGGIHLPTDALSK_MS2. Figure S3.-B Dot-product of a given peptide of gi351724717refNP-001237323-1_GLFEGGIHLPTDALSK_MS2. Figure S3.-C MRM chromatogram of a given peptide of gi351724717refNP-001237323-1_ GLFEGGIHLPTDALSK_MS2. (ZIP 197 kb) [file 12864_2017_3825_MOESM2_ESM.zip › Fig.S2-C.tif]

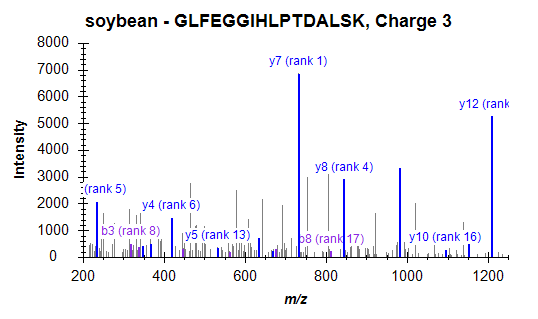

Supplement: Supplementary file 2 — Correlation between predicted retention time and observed retention time. Figure S2.-A MS/MS spectrum of a given peptide of gi351723671refNP-001237543-1_WVAFVDNEIQK_MS2. Figure S2.-B Dot-product of a given peptide of gi351723671refNP-001237543-1_WVAFVDNEIQK_MS2. Figure S2.-C MRM chromatogram of a given peptide of gi351723671refNP-001237543-1_WVAFVDNEIQK_MS2. Figure S3-A MS/MS spectrum of a given peptide of gi351724717refNP-001237323-1_GLFEGGIHLPTDALSK_MS2. Figure S3.-B Dot-product of a given peptide of gi351724717refNP-001237323-1_GLFEGGIHLPTDALSK_MS2. Figure S3.-C MRM chromatogram of a given peptide of gi351724717refNP-001237323-1_ GLFEGGIHLPTDALSK_MS2. (ZIP 197 kb) [file 12864_2017_3825_MOESM2_ESM.zip › Fig.S3-A.tif]

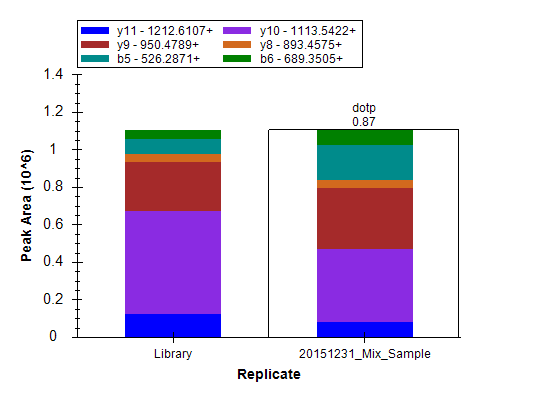

Supplement: Supplementary file 2 — Correlation between predicted retention time and observed retention time. Figure S2.-A MS/MS spectrum of a given peptide of gi351723671refNP-001237543-1_WVAFVDNEIQK_MS2. Figure S2.-B Dot-product of a given peptide of gi351723671refNP-001237543-1_WVAFVDNEIQK_MS2. Figure S2.-C MRM chromatogram of a given peptide of gi351723671refNP-001237543-1_WVAFVDNEIQK_MS2. Figure S3-A MS/MS spectrum of a given peptide of gi351724717refNP-001237323-1_GLFEGGIHLPTDALSK_MS2. Figure S3.-B Dot-product of a given peptide of gi351724717refNP-001237323-1_GLFEGGIHLPTDALSK_MS2. Figure S3.-C MRM chromatogram of a given peptide of gi351724717refNP-001237323-1_ GLFEGGIHLPTDALSK_MS2. (ZIP 197 kb) [file 12864_2017_3825_MOESM2_ESM.zip › Fig.S3-B.tif]

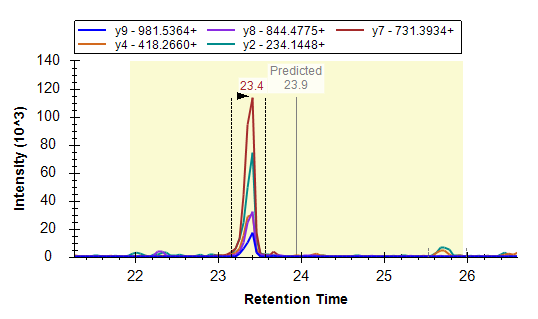

Supplement: Supplementary file 2 — Correlation between predicted retention time and observed retention time. Figure S2.-A MS/MS spectrum of a given peptide of gi351723671refNP-001237543-1_WVAFVDNEIQK_MS2. Figure S2.-B Dot-product of a given peptide of gi351723671refNP-001237543-1_WVAFVDNEIQK_MS2. Figure S2.-C MRM chromatogram of a given peptide of gi351723671refNP-001237543-1_WVAFVDNEIQK_MS2. Figure S3-A MS/MS spectrum of a given peptide of gi351724717refNP-001237323-1_GLFEGGIHLPTDALSK_MS2. Figure S3.-B Dot-product of a given peptide of gi351724717refNP-001237323-1_GLFEGGIHLPTDALSK_MS2. Figure S3.-C MRM chromatogram of a given peptide of gi351724717refNP-001237323-1_ GLFEGGIHLPTDALSK_MS2. (ZIP 197 kb) [file 12864_2017_3825_MOESM2_ESM.zip › Fig.S3-C.tif]
